# Eliminating primer dimers and improving SNP detection using self-avoiding molecular recognition systems

**DOI:** 10.1093/biomethods/bpaa004

**Published:** 2020-02-10

**Authors:** Zunyi Yang, Jennifer T Le, Daniel Hutter, Kevin M Bradley, Benjamin R Overton, Chris McLendon, Steven A Benner

**Affiliations:** b1 Foundation for Applied Molecular Evolution (FfAME), 13709 Progress Blvd, Box 7, Alachua, FL 32615, USA; b2 Firebird Biomolecular Sciences LLC, 13709 Progress Blvd, Box 17, Alachua, FL 32615, USA

**Keywords:** multiplex PCR, primer dimer, SNP detection, allele-specific PCR, diagnostics

## Abstract

Despite its widespread value to molecular biology, the polymerase chain reaction (PCR) encounters modes that unproductively consume PCR resources and prevent clean signals, especially when high sensitivity, high SNP discrimination, and high multiplexing are sought. Here, we show how “self-avoiding molecular recognition systems” (SAMRS) manage such difficulties. SAMRS nucleobases pair with complementary nucleotides with strengths comparable to the A:T pair, but do not pair with other SAMRS nucleobases. This should allow primers holding SAMRS components to avoid primer–primer interactions, preventing primer dimers, allowing more sensitive SNP detection, and supporting higher levels of multiplex PCR. The experiments here examine the PCR performances of primers containing different numbers of SAMRS components placed strategically at different positions, and put these performances in the context of estimates of SAMRS:standard pairing strengths. The impact of these variables on primer dimer formation, the overall efficiency and sensitivity of SAMRS-based PCR, and the value of SAMRS primers when detecting single nucleotide polymorphisms (SNPs) are also evaluated. With appropriately chosen polymerases, SNP discrimination can be greater than the conventional allele-specific PCR, with the further benefit of avoiding primer dimer artifacts. General rules guiding the design of SAMRS-modified primers are offered to support medical research and clinical diagnostics products.

## Introduction

Despite its widespread value to molecular biologists, the polymerase chain reaction (PCR) [1, 2] has an “endless ability to confound” [3]. Considerable classical work sought to improve its reliability, seeking to eliminate bias, cross-reactions, and other artifacts [4–7]. One goal of this work is to manage the well-recognized problematic formation of primer dimers [8–12], which can consume PCR resources, including the polymerase, primers, and the triphosphates, as well as downstream sequencing resources. This consumption becomes worse as target molecules become more numerous and scarcer [13]. High concentrations of primers encourage off-target interactions, amplification of short primer dimers is more efficient than amplification of the desired amplicon, and primer–primer interactions eventually eliminate target amplification entirely [14, 15].

Algorithms for designing primer sets with minimal cross-reactivity and intramolecular hairpin formation are available [16–19]. However, because they capture only imperfectly the actual biophysics, the primers that they design must be confirmed by experiment, especially as the level of multiplexing increases [20].

Various “hot start” methods have been developed to mitigate primer dimer formation, under a design where primer–primer pairs are weaker than the primer–target pairs [21]. Hot start strategies withhold an essential component of the PCR until the temperature is raised to a point where primer–primer duplexes and primer off-target duplexes have melted [22]. The withheld component may be the DNA polymerase [23, 24], the primers [25, 26], the dNTPs [27], or magnesium [28]. However, none of these totally eliminate primer dimer formation. Further, protection via a hot start is lost after the first denaturing step. This means that primer dimers can still be formed and propagated in the second and later stages of annealing and amplification.

The waste of resources via primer dimer formation can also be lowered by physically separating primers on a solid support or within an emulsion [29, 30]. However, the amplification efficiency is generally reduced. Other methods, such as touch down PCR [31], nested PCR [32], and digital PCR [33, 34] are also used to suppress the formation of primer dimer. While the nature of primer interactions is unchanged in these alternative methods, primer dimers still form to some extent.

**Figure 1:**
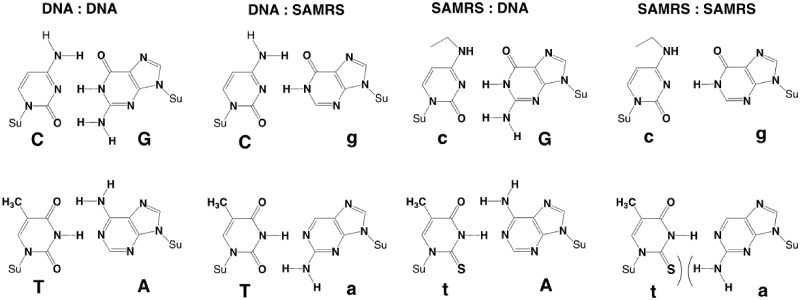
Chemical structures of the self-avoiding molecular recognition system (SAMRS). Pairs between standard nucleobases (left, DNA:DNA). Pairs between standard nucleobases and their SAMRS complements (**g**, **c**, **a**, and **t**, middle, DNA:SAMRS and SAMRS:DNA). Pairs between SAMRS nucleobases and their formal SAMRS complements; these do not contribute to duplex stability (right, SAMRS:SAMRS). Su, sugar backbone.

A still different approach to mitigate primer dimer formation changes the structure of the DNA itself. For example, self-avoiding molecular recognition systems (SAMRS) [35] replace the standard nucleobases (G, A, C, and T) with alternative nucleobases (**g**, **a**, **c**, and **t**; Fig. 1) by strategically adjusting the hydrogen bonding moieties. Once adjusted, **g** continues to pair with C, **c** with G, **a** with T, and **t** with A. However, SAMRS:SAMRS pairs (**a:t** and **g:c**) are weak. Thus, **gact** SAMRS primers are expected to anneal to their natural CTGA DNA targets, allowing them to serve as primers for a forward PCR reaction and serve as a template for a reverse PCR reaction [35]. However, primer–primer interactions should be significantly decreased.

Because each SAMRS:standard pair is effectively joined by only two hydrogen bonds, the binding strengths of oligonucleotide primers that contain SAMRS:standard pairs are weaker than primers with standard:standard pairs, especially GC rich pairs. This means that primers modified with SAMRS components anneal, extend, and template less efficiently and with a longer specificity “footprint” than the standard primers under identical conditions [36, 37]. Therefore, the number of SAMRS components in a primer must be limited, and the position of modification must be optimized when designing SAMRS primers.

This prompts two general design questions whose answers prove to have practical value: How “many” SAMRS nucleotides must be incorporated into a primer for optimal benefit? Second, “where” should SAMRS components be placed to support efficient PCR with less primer dimer formation?

We answer these questions here via a detailed study of SAMRS primers in PCR. This study offers rules for the optimal use of SAMRS components in PCR in general. These are set in the context of melting temperature studies that may give heuristics to design SAMRS-containing PCR primers. These are compared to and combined with literature that examined related “pseudo-complementary” pairs [38, 39]. We then evaluate the SAMRS components individually, and the extent to which they diminish primer dimer formation and slow the rate of PCR. Finally, we identify SAMRS design rules to better discriminate single-nucleotide polymorphisms (SNPs) in template targets and facilitate faster development of multiplexed PCR by using SAMRS primers.

## Materials and methods

### Oligonucleotides

SAMRS-containing oligonucleotides were synthesized on ABI 394 and ABI 3900 instruments using standard phosphoramidite chemistry. All SAMRS phosphoramidites were from Glen Research or ChemGenes. No changes were needed for coupling and deprotection of SAMRS components compared with standard phosphoramidites (dmf-dG, Ac-dC, and Bz-dA, dT) as recommended by synthesizer manufacturer. For in-house tests, SAMRS-containing oligonucleotides were synthesized either DMT-on or DMT-off. The DMT-off oligonucleotides were deprotected in aqueous ammonium hydroxide (28–33% NH_3_ in water) at 55°C overnight (10–12 h), purified by ion-exchange HPLC (Dionex DNAPac PA-100, 22 × 250 mm column), and desalted over SepPak cartridges. Oligonucleotides synthesized DMT-on were deprotected with ammonia as above, followed by removal of the DMT group using Glen-Pak Cartridges; if the resulting purity (by analytical ion-exchange HPLC) was below 80%, then that oligonucleotide was further purified by preparative ion-exchange HPLC. The purity of each oligonucleotide was analyzed again by analytical ion-exchange HPLC. Oligonucleotides with only natural nucleobases were obtained from Integrated DNA Technologies (IDT). For diagnostic kits, all SAMRS-containing oligonucleotides were synthesized via the DMT-off strategy and purified by ion-exchange HPLC to meet the purity standard set by the diagnostic kits (e.g. >85% or 90%).

### Melting temperature analysis

The melting temperatures (T_m_s) of duplexes containing single SAMRS:standard and SAMRS:SAMRS pairs were obtained from the literature [35] (Supplementary Fig. S1). As a full set of thermodynamic parameters would require melting studies of oligonucleotides that are too short to serve efficiently as primers, we expanded on this literature using a heuristic approach.

Experimentally, the T_m_s of standard oligonucleotide duplexes and the corresponding SAMRS:standard duplexes were measured in PCR buffer of JumpStart *Taq* DNA polymerase (1 µM of each oligonucleotide, 10 mM Tris-HCl, 50 mM KCl, pH 8.3 at 25°C, 1.5 mM, or 5.0 mM of MgCl_2_). The oligonucleotides had the following sequences (upper case letters G, A, C, T, and N indicate standard nucleobases; lower case bold letters **g**, **a**, **c**, **t**, and **n** indicate SAMRS components; the standard complementary sequences are not shown):

Set 1 sequence: 5′-GAG CTG AGG TCA GTG T **n n n n** C-3′

Set 2 sequence: 5′-GAG CTG AGG TCA GTG N **n a t n** N-3′

Set 3 sequence: 5′-GCT CGA ATT GCA CCC T **n n n n** C-3′

The melting curves were visualized using fluorescent dye (0.5× EvaGreen) in a Roche LightCycler^®^ 480 with the following temperature profile: (i) denature and anneal duplexes: 95°C for 3 min, cool to 40°C with melting-curve setting (10 acq/°C; ∼4–5°C/min), heat again to 50°C and hold for 10 min; (ii) slowly denature duplexes from 50°C to 90°C with melting-curve setting (100 acq/°C; ∼1°C/min). Each set of duplexes was measured 3 times. Standard:standard and SAMRS:standard duplexes were run in parallel on the same 96-well plate. T_m_s were obtained from the slow denaturing ramps (ii) using the automatic calculation method of the Roche LightCycler (MeltFactor set at 1.2, QuantFactor set at 20). ΔT_m_ values were calculated in Microsoft Excel for each ramp individually (Supplementary Tables S1-1, S1-2, and S1-3).

We then analyzed the sequences to find heuristically the best adjustments for each nearest neighbor pair that would most closely match the T_m_ deltas obtained experimentally from 84 sequences (Supplementary Tables S1-1 and S1-2). Code broke each sequence into nearest-neighbor doublets (e.g. T**cata**T is broken into the doublet: [T**c**] [**ca**] [**at**] [**ta**], and [**a**T]). An initial T_m_ “effect estimate” was iteratively applied to all 48 possible doublets. The average difference between the input T_m_ deltas and those calculated from an initial set of estimates was recorded. The program then adjusted the T_m_ of randomly chosen doublets in 0.1 increments (adding 0.1 from the first and subtracting 0.1 from the other). If the adjustment improved the correspondence of the effect estimate and the data, the adjustment was retained. If it did not, the opposite adjustment was attempted (subtracting 0.1 from the first and adding 0.1 from the other). Adjustments that gave improvements were retained; the others were discarded. This process was continued until no improvements were found after 50 iterations through all doublets. Then, the process was repeated until no better estimates could be found in 20 000 attempts.

### Evaluation of amplification efficiency of 256 SAMRS-containing primers by real-time PCR

For real-time PCR, only the reverse primer contained SAMRS components; a common standard forward primer was used for all 256 reverse SAMRS primers. The primers were designed so as to be about 20 nucleotides in length. So that all primers had approximately equal T_m_ values (∼60°C), their lengths were adjusted by adding up to three nucleobases at the 5′-end (5′- (GCT) CGA ATT GCA CCC T **n n n n** C-3′; the bases in parentheses were added to adjust T_m_ using the heuristic parameters obtained above).

A degenerate template was designed where the template sites that complement the SAMRS segment of the reverse primers were comprised of mixed standard nucleotides (*N* = A:G:C:T at 1:1:1:1:1 ratio), meaning that the SAMRS-containing primers were perfectly matched to the template at 1/256 of the total template concentration in the PCR mixture. The template sequence was further designed to avoid hairpin structures, particularly near the 3′-ends of the primer binding sites. The designed degenerate template was synthesized by IDT, with standard desalting as the only downstream manipulation, to avoid changes in the relative amounts of the mixture components during purification.

5′-TAC GGC TAT GGA CAT CAC-3′

5′-TAC GGC TAT GGA CAT CAC ATTCAGCGCAAATCAGGTAAG G **N N N N** A GGG TGC AAT TCG AGC-3′

3′-C **n n n n** T CCC ACG TTA AGC (TCG)-5′

The common forward primer (0.2 µM), one of the reverse SAMRS primers (1 out of 256 reverse primers, 0.2 µM), and degenerate template (10 pM total, each individual template at ∼40 fM) were mixed with dNTPs (each 0.2 mM), Hot Start JumpStart *Taq* DNA polymerase (0.05 units/µl, Sigma), and EvaGreen dye (1×, Biotium) in 1× PCR buffer (10 mM Tris-HCl, 50 mM KCl, pH 8.3 at 25°C, 1.5 or 5.0 mM MgCl_2_). Real-time PCRs were performed in triplicate on the Roche LightCycler 480 instrument using the following temperature program: 95°C for 4 min, followed by 40 cycles (95°C for 30 s, 60°C for 120 s, and 72°C for 60 s, measure fluorescence at 72°C), followed by melting curve analysis. LightCycler 480 software was used to obtain a cycle threshold (Ct). Results are reported in Supplementary Table S2.

The T_m_s of additional sets of oligonucleotides having different numbers (1–4) and placements of SAMRS components, with various lengths (23–30 nt), and different overall G:C:A:T ratios were also tested (Supplementary Table S3). Here, T_m_s were obtained in 1× KlenTaq buffer [1 µM of each oligo, 50 mM of Tris (pH 8.3), 0.25 µg/µl of BSA, 1× LCGreen, and 3 mM MgCl_2_]. The melting curves were visualized using fluorescent dye (LCGreen, Biofire Defence) by the Roche LightCycler^®^ 480 with the following temperature profile: (i) denature and anneal duplexes: 95°C for 1 min, 50°C for 5 min; (ii) slowly denature duplexes from 50°C to 90°C (3.6°C/min) and continually monitor fluorescent signal (10 acquisitions/°C). Each set of duplexes was measured 3 times. These SAMRS-containing oligos were further tested in PCR and their performance was compared to the standard oligos.

### Prevention of primer dimer in the worst-case pair of primers

**Figure 2:**
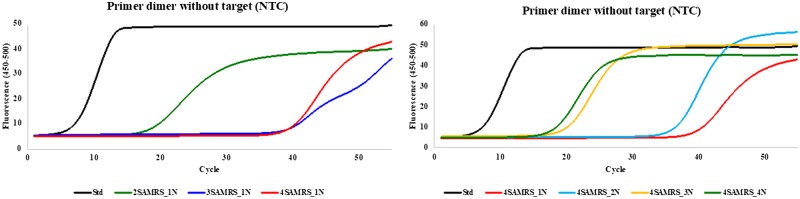
PCR assay with forward and reverse primers having six perfectly matched base pairs at their 3′-ends with no target (3 mM Mg^2+^). Left: Results from primers having different numbers (0, 2, 3, and 4) of SAMRS components: 2SAMRS, 3SAMRS, and 4SAMRS have 2, 3, and 4 SAMRS components, respectively; Std (black) primers have no SAMRS components. Note the ability of SAMRS components to delay the appearance of primer dimers. Right: Results from primers having four SAMRS components (all 4SAMRS) placed 1, 2, 3, and 4 nucleotides away from the 3′-end of primers (1N, 2N, 3N, and 4N, respectively). Note the decreasing ability of SAMRS to prevent primer dimer formation with greater distance from the 3′-end of the primer.

**Figure 3:**
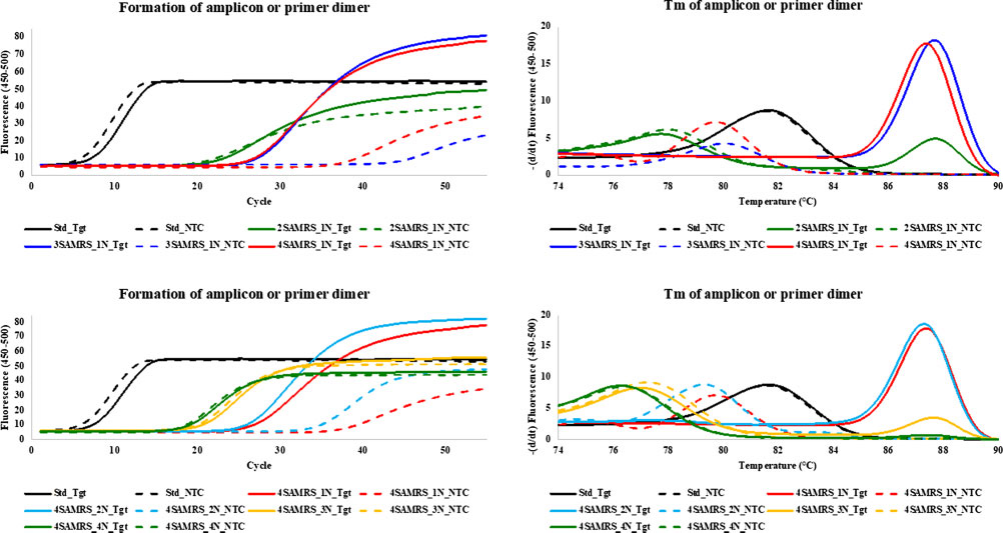
PCR assay with forward and reverse primers having six perfectly matched base pairs at their 3′-ends. Curves are shown in the presence of target (lines marked Tgt, solid) and absence of target (lines marked NTC, dashed). All reactions were at 3 mM Mg^2+^. The numbers of SAMRS components (2, 3, or 4) are indicated by 2SAMRS, 3SAMRS, and 4SAMRS. The positions of four SAMRS components (4SAMRS) are placed 1, 2, 3, and 4 nucleotides away from the 3′-end of primers (1N, 2N, 3N, and 4N, respectively). Left: PCR amplification curves. Right: Melts curves after 55 cycles. The melts show that the 3SAMRS-1N, 4SAMRS-1N, and 4SAMRS-2N primers (blue, red, and aqua solid lines) produced only amplicons (T_m_ ≈ 87.5°C) in the presence of the target. The 2SAMRS-1N and 4SAMRS-3N primers (green and yellow solid lines) gave a mix of amplicon and primer dimer, while standard primers only generated primer dimer (black, T_m_ ≈ 81.5°C).

A “worst case” pair of primers were tested with or without target in parallel PCR (20 µl). Standard forward and reverse primers (Std-Fp-25 and Std-Rp-25, 1 µM each) having six perfectly matched base pairs at their 3′-ends, SAMRS forward and reverse primers containing different numbers and positions of SAMRS components (2SAMRS-1N, 3SAMRS-1N, 4SAMRS-1N, 4SAMRS-2N, 4SAMRS-3N, and 4SAMRS-4N, 1 µM each) were tested in PCR containing 1× reaction buffer (0.25 µg/µl of BSA, 50 mM of Tris, pH 8.3), MgCl_2_ (2, 3, or 4 mM), dNTPs (each 0.2 mM), 1× LCGreen^®^ Plus (Biofire Defense), template (10^4^ copies per assay or H_2_O as NTC), and 0.8 units of KlenTaq1 DNA polymerase (0.04 U/µl, AB Bioscience). The Hot Start KlenTaq1 was achieved by Anti-Taq Monoclonal Antibody (Clone 8C1, 8.8 ng/µl). Real-time PCRs were performed under identical conditions: 95°C for 2 min, 55 cycles (95°C for 10 s, 55°C for 10 s, and 72°C for 12 s) followed by melting curves analysis in the Roche LightCycler^®^ 480 PCR system. LightCycler^®^ 480 software was used to calculate Ct and T_m_s. Each assay had three repeats (Tables 1 and 2, Figs 2 and 3, Supplementary Fig. S2).

**Table 1:** Formation of primer dimer in no target control (NTC) with a “worst case” pair of primers

	Ct of primer dimer formation			
Primer types	Mg^2+^ (2 mM)	Mg^2+^ (3 mM)	Mg^2+^ (4 mM)	Average Ct
Std primers	12.6	7.0	4.0	7.9
2SAMRS-1N	29.3	18.9	15.6	21.3
3SAMRS-1N	48.1	44.6	42.2	45.0
4SAMRS-1N	50.0	40.0	37.4	42.5
4SAMRS-2N	52.0	36.6	33.5	40.7
4SAMRS-3N	27.2	20.1	17.7	21.7
4SAMRS-4N	25.2	18.1	15.5	19.6

**Table 2:** Formation of amplicon and primer dimer in the presence of target and no target with a “worst case” pair of primers

	Ct of amplicon or primer dimer formation		Average ΔCt
Primer types	With target (10^4^ copies)	Without target (NTC)	Ct (NTC) − Ct (target)
Std primers	7.6^a^	6.0^a^	−1.6
2SAMRS-1N	22.7	20.9	−1.8
3SAMRS-1N	27.8	44.5	16.7
4SAMRS-1N	27.2	37.7	10.5
4SAMRS-2N	26.6	35.0	8.4
4SAMRS-3N	20.8	20.1	−0.7
4SAMRS-4N	18.8	18.1	−0.7

a Based on melting temperature (Fig. 3, right), these are all primer dimers at 3 mM of Mg^2+^.

### SAMRS-containing primers support efficient PCR, suppress primer dimer, and improve SNPs discrimination

A “least-worst case” pair of primers was designed to target the conserved regions of the reverse transcriptase gene in HIV1 (subtype B). The PCR efficiency, primer dimer formation, and SNP discrimination were compared between standard primers and primers modified with one up to four SAMRS components. PCR (10 µl) containing a common standard forward primer (HIV-Std-Fp, 0.5 µM) and a reverse primer modified with different numbers of SAMRS components (HIV-Std-Rp, 1SAMRS, 2SAMRS, 3SAMRS, or 4SAMRS, each 0.5 µM), or a common standard reverse primer (HIV-Std-Rp, 0.5 µM) and a forward primer modified with different numbers of SAMRS components (each 0.5 µM), were performed in 1× reaction buffer (0.25 µg/µl of BSA, 50 mM of Tris, pH 8.3), MgCl_2_ (3 mM), dNTPs (each 0.2 mM), 1× or 0.5× LCGreen^®^ Plus (Biofire Defense), HIV-allele-A/G-template (10^4^ copies of cDNA per assay or H_2_O as NTC), 0.4 units of KlenTaq1 DNA polymerase (0.04 U/µl, AB Bioscience) plus Anti-Taq Monoclonal Antibody (Clone 8C1, 8.8 ng/µl). Real-time PCRs were performed under identical conditions: 95°C for 2 min, 60 cycles (95°C for 10 s, 60°C for 12 s, and 72°C for 12 s) followed by melting curves analysis in the Roche LightCycler^®^ 480 PCR system. LightCycler^®^ 480 software was used to calculate Ct and T_m_s. Each assay had three repeats (Tables 3 and 4, Supplementary Table S4). Other DNA polymerases, such as HiDi^®^ DNA polymerase (0.05 U/µl, myPOLS Biotec), AmpliTaq God (0.1 U/µl, ThermoFisher) and SNPase HotStart DNA Polymerase (0.05 U/µl, Bioron) were also tested in the manufacture recommended buffer with adjusted Mg^2+^ at 3 mM and 0.2× EvaGreen (Fisher Scientific).

**Table 3:** Ct and ΔCt values of PCR amplifications using reverse primer with different numbers of SAMRS components with KlenTaq1 DNA polymerase

Allele-specific PCR using KlenTaq1 DNA polymerase				
Forward primer	Reverse primer	Match C:G (ΔCt)	Mismatch C:A (ΔCt)	NTC (ΔCt)
Common HIV-Std-Fp	HIV-Std-Rp	29.8 (0) reference	32.1 (2.3)	36.1 (6.3)
	Std-1Mis C to *T*	30.1 (0.3)	39.8 (dimer)	36.7 (6.9)
	1SAMRS-Tc-1N	29.8 (0)	34.6 (4.8)	37.0 (7.2)
	2SAMRS-tc-1N	30.1 (0.3)	35.4 (5.6)	42.9 (13.1)^a^
	3SAMRS-atc-1N	31.1 (1.3)	40.2 (10.4)	55 (25.2)^a^
	4SAMRS-gatc-1N	32.9 (3.1)	42.9 (13.1)	NA
	2SAMRS-aTc-1N	30.5 (0.7)	37.2 (7.4)	53.9 (24.1)^a^
	3SAMRS-gaTc-1N	31.7 (1.9)	38.8 (9)	NA

**Table 4:** Ct and ΔCt values of PCR amplifications using reverse primer with different numbers of SAMRS components with HiDi DNA polymerase

Allele-specific PCR using HiDi DNA polymerase				
Forward primer	Reverse primer	Match C:G (ΔCt)	Mismatch C:A (ΔCt)	NTC (ΔCt)
Common HIV-Std-Fp	HIV-Std-Rp	29.7 (0) reference	37.4 (7.7)	NA
	Std-1Mis (C to *T*)	33.8 (4.1)	37.0 (dimer)^a^	49.0 (19.3)
	1SAMRS-Tc-1N	28.8 (−0.9)	55.8 (26.1)	NA
	2SAMRS-tc-1N	30.6 (0.9)	51.5 (21.8)	NA
	3SAMRS-atc-1N	33.1 (3.4)	NA	NA
	4SAMRS-gatc-1N	36.4 (6.7)	NA	NA

A “least-worst case” pair of primers was tested in allele-specific PCR (60 cycles). All PCR have three replicates for matched template G, mismatched template A, and NTC.

^a^One-third of replicates show amplification signal. Dimer indicates primer dimer. NA indicates No Amplification.

A set of four synthetic templates with a single base mutation (A, G, C, or T) was amplified by a common standard forward primer (Common Std-Fp, 0.5 µM), an allele-specific standard reverse primer (Std-Rp-T-allele or Std-Rp-C-allele, each 0.5 µM) and a set of reverse primers modified with SAMRS components in different positions and numbers (each 0.5 µM). All PCR (10 µl) were performed 1× reaction buffer (0.25 µg/µl of BSA, 50 mM of Tris, pH 8.3), MgCl_2_ (3 mM), dNTPs (each 0.2 mM), 0.5× LCGreen^®^ Plus (Biofire Defense), allele-A/G/C/T-template (10^4^, 10^3^, 10^2^ copies per assay or H_2_O as NTC), with 0.5 units of HiDi DNA polymerase (0.05 U/µl), or 0.4 units of KlenTaq1 DNA polymerase (0.04 U/µl) plus Anti-Taq Monoclonal Antibody (8.8 ng/µl). Real-time PCRs conditions: 95°C for 2.5 min, 60 cycles (95°C for 10 s, 60°C for 10 s, and 72°C for 12 s), or 95°C for 2.5 min, 60 cycles (95°C for 10 s, 65°C for 15 s), followed by melting curves analysis in the Roche LightCycler^®^ 480 PCR system. LightCycler^®^ 480 software was used to calculate Ct and T_m_s. Each assay had either three or six repeats (Tables 5–7, Supplementary Tables S5 and S6).

**Table 5:** Ct and ΔCt values of PCR amplifications using reverse primer with different numbers and positions of SAMRS components with KlenTaq1 DNA polymerase

Ct and ΔCt of SNP discrimination using KlenTaq1 DNA polymerase							
Forward primer	Reverse primer	Match T:A	Mismatch T:G	Mismatch T:C	Mismatch T:T	Average (ΔCt)	NTC
Common Std-Fp	Std-Rp-T-allele	28.3 (0) reference	31.3 (3)	33.1 (4.8)	33.5 (5.2)	4.4	NA
	2SAMRS-Tgt-1N	29.5 (1.2)	34.5 (6.2)	37.3 (9)	37.2 (8.9)	8.1	NA
	3SAMRS-tgt-1N	29.9 (1.6)	36.1 (7.8)	38.5 (10.2)	38.6 (10.3)	9.5	NA
	4SAMRS-ctgt-1N	29.7 (1.4)	37.7 (9.4)	45.5 (17.2)	38.8 (10.5)	12.4	NA
	4SAMRS-actg-2N	30.1 (1.8)	38.5 (10.2)	42.1 (13.8)	40.1 (11.8)	12.0	NA
	4SAMRS-tact-3N	29.6 (1.3)	36.0 (7.7)	38.6 (10.3)	37.7 (9.4)	9.2	NA
	4SAMRS-ttac-4N	29.5 (1.2)	35.0 (6.7)	37.1 (8.8)	38.1 (9.8)	8.4	NA

**Table 6:** Ct and ΔCt values of PCR amplifications using reverse primer with different numbers and positions of SAMRS components with HiDi DNA polymerase

	Ct and ΔCt of SNP discrimination using HiDi DNA polymerase					
Forward primer	Reverse primer	Match T:A	Mismatch T:G	Mismatch T:C	Mismatch T:T	NTC
Common Std-Fp	Std-Rp-T-allele	29.1 (0) reference	38.1 (9)	NA	NA	NA
	2SAMRS-Tgt-1N	31.7 (2.6)	41.8 (12.7)^a^	NA	NA	NA
	3SAMRS-tgt-1N	33.1 (4.0)	NA	NA	47.3 (18.2)^a^	NA
	4SAMRS-ctgt-1N	33.8 (4.7)	NA	NA	NA	NA
	4SAMRS-actg-2N	34 (4.9)	NA	NA	NA	NA
	4SAMRS-tact-3N	32.5 (3.4)	43.9 (14.8)^a^	NA	NA	NA
	4SAMRS-ttac-4N	32.1 (3.0)	41.8 (12.7)^a^	NA	NA	NA

All allele-specific PCR have three replicates for matched template A, mismatched templates (G, C, and T), and NTC.

^a^One-third of replicates show amplification signal. NA indicates No Amplification.

**Table 7:** Compare PCR efficiency and sensitivity of standard primers with SAMRS primers

Allele-specific PCR using HiDi DNA polymerase								
Matched or mismatched template		10^4^ copies		10^3^ copies		10^2^ copies		NTC
Forward primer	Reverse-T-allele	Match T:A	Mismatch T:G	Match T:A	Mismatch T:G	Match T:A	Mismatch T:G	
Std-Fp	Std-Rp	27.5 (0.0)	35.8 (8.3)	32.0 (4.5)	38.9 (11.4)	34.8 (7.3)	50.6 (23.1)^b^	38.6 (dimer)^c^
2SAMRS-Fp	2SAMRS-Rp	29.5 (2.0)	39.9 (12.4)	33.8 (6.3)	42.9 (15.4)	37.7 (10.2)	55 (27.5)^b^	42.5 (dimer)^a^

All allele-specific PCR have six replicates for matched template A, mismatched templates G, and NTC.

^a^One-sixth,

^b^two-sixths, and

^c^three-sixths of replicates generated amplification signal. Dimer indicates primer dimer.

## Results and discussion

The optimal use of SAMRS (**a **=** **2-aminopurine; **t **=** **2-thiothymine, **c** = N^4^-ethyl-5-cytosine, and **g** = inosine) involves managing a principal feature of SAMRS primers: having fewer/weaker hydrogen bonding that lowers their affinity to a natural template. This implies that the number of SAMRS modifications in primers should be limited. Conversely, introducing too few SAMRS components does not completely prevent the formation of primer dimers and other products arising from undesired primer–primer interactions.

Here, we regularized the search for the optimum tradeoff between these two by exploring three variables:

- The number of SAMRS components in the primer. Here, we explored primers containing 1, 2, 3, or 4 SAMRS components.
- The position of the SAMRS components in the primer relative to the 3′-extendable end of the primer. Here, we explored placement of the SAMRS component at the penultimate nucleotide at 3′-end of the primer (Position 2), or further back in the primer (Positions 3, 4, 5 …), where the 3′-nucleotide is in Position 1, and is always a standard nucleotide.
- The introduction of identical SAMRS components at two or more consecutive positions.

We then explored these variables with both melting temperature and PCR studies.

### Melting temperature analysis of the oligonucleotides containing SAMRS components

The literature studies with single matched and mismatched SAMRS:SAMRS and SAMRS:standard pairs (X:Y) at the center of the duplex (Supplementary Fig. S1) report that **c**:G and **t**:A pairs contribute essentially the same to duplex stability as the A:T pair, while **a**:T pair contributes about 2°C less than the A:T pair. However, **c**:**g**, **g**:**c**, **t**:**a**, and **a**:**t** pairs are about 7.5°C, 6.8°C, 3.7°C and 3.5°C less than the C:G, G:C, T:A, and A:T pairs, respectively (Supplementary Fig. S1). Further, **g** (as inosine) had less mismatch discrimination; the difference between **g**:C and **g**:A is about 3°C. This comports with the view that inosine is an imperfect “universal base,” able to pair with C > A>T > G [40]. In contrast, SAMRS **c**, **a,** and **t** have excellent mismatch discrimination (Supplementary Fig. S1).

Separately, Gamper *et al.* explored a similar pairing in their “pseudocomplementary” DNA [38, 39]. When literature data are combined, the impact of incorporating a SAMRS:standard pair (relative to a standard pair) are represented by these deltas: **g**:C (−4.5°C ± 0.5°C), **g**:A (−9.0°C ± 0.2 °C), and **g**:T (−11.3°C ± 1.3 °C) pairs are less stable than G:C; **c**:G (−3.0°C ± 0.5 °C) and **c**:A (−15.0°C ± 2 °C) are less than C:G; **a**:T (−1.0°C ± 0.4°C) and **a**:C (−7.2°C ± 0.2°C) are less than A:T; while, **t**:A (+0.8°C ± 0.4°C) pair is more stable than T:A and **t**:G (−5.8°C ± 0.2°C) is less than T:A.

The variances reflect the impact of the surrounding sequence (“context”) on the contribution of specific SAMRS:standard pairs to overall duplex stability. Further, the difference between a SAMRS:standard pair and the analogous standard pair is larger for **g** and **c** (about −4°C ± 0.5°C) than for **a** and **t** (about 0°C ± 0.4°C). This is consistent with the fact that the G:C pair has three hydrogen bonds, while the A:T pair has only two.

We then tested a larger set of duplexes containing 18, 21, 23, 25, 27, and 30 base pairs with different overall G:C:A:T ratios. The data set included measurements of T_m_s for a subset of possible combinations of four SAMRS sequences (Supplementary Table S1). An additional data set was collected for duplexes having different numbers (1–4) and placements of SAMRS components (Supplementary Table S3).

These new results are consistent with literature data, and suggest “rules” that are summarized as follows:

1. The deltas depend on the specific SAMRS sequences.

1.1 The average T_m_ of standard:standard duplexes (Set 1, NNNN:NNNN) is 70.5°C ± 2.3°C (1.5 mM Mg^2+^).
1.2 The average T_m_ of SAMRS:standard duplexes (Set 1, **nnnn**:NNNN) is 66.5°C ± 1.2°C, and a decrease of 4.0°C ± 2.7°C (Supplementary Table S1-1).
1.3 Adding **g**- and **c**-rich SAMRS sequences (e.g. **gcgc**, **gggg**, **gccg**, and **cccc**) gives a larger T_m_ change (−10.6°C, −9.1°C, −9.0°C, and −7.6°C, respectively).
1.4 Adding **t**-rich sequences (e.g. **tttt**) “increases” T_m_ by 2.6°C.
1.5 Adding **a**/**t** rich SAMRS sequences (e.g. **taat** and **tata**) has little impact on T_m_ (±0.3°C).
1.6 Adding mixed SAMRS sequences (e.g. **acgt**, **catg, gtac**, and **caca**) lowers T_m_ by ∼4°C.

2. Nevertheless, T_m_ range is desirably smaller with SAMRS sequences than with standard sequences. The T_m_s of standard:standard duplexes range from the most stable (GCCG, T_m_ = 75.5°C) to the least stable (TATA, T_m_ = 66.5°C), a range of 9°C. The T_m_s of SAMRS:standard duplexes range from the most stable (**tttt**:AAAA, T_m_ = 69.9°C) to the least stable duplex (**gcgc**:CGCG, T_m_ = 63.7°C), a range of 6.2°C.
3. Increasing Mg^2+^ concentration (from 1.5 to 5 mM) increases the T_m_s of both standard and SAMRS:standard duplexes comparably (averaging 1.2°C and 1.3°C, respectively).
4. Increasing Mg^2+^concentration appears to make **g**- and **c**-rich SAMRS sequences (**gcgc** and **gggg**) pair with their standard complements more like standard sequences (GCGC and GGGG; Supplementary Table S1-1).

To develop heuristics that capture context dependence, the second set of duplexes was examined (Set 2, Supplementary Table S1-2). Here, the two middle nucleotides in the SAMRS segment were constrained to be **a** and **t**, which have little effect on ΔT_m_ in the sequence (N **n a t n** N); T_m_s were compared with those of the corresponding standard sequence (N N A T N N). The average T_m_ of SAMRS:standard duplexes are 1.9°C ± 1.7°C lower than the standard duplexes (Supplementary Table S1-2). This suggests heuristically the destabilization of a duplex containing SAMRS components is proportional to the number of SAMRS modifications and the percentage of **g** and **c**.

To further explore context dependence, the third set of sequences (Set 3, Supplementary Table S1-3) was tested. Here, the T_m_s of SAMRS oligos and the average ΔT_m_ between standard and SAMRS oligos (5.3°C ± 3.0°C) confirmed the interpretations drawn from Set 1 studies. Again, **t**-rich SAMRS sequences increase T_m_ by ∼0.7°C, **aatt** has little effect (−0.3°C), and **g**- and **c**-rich sequences decrease T_m_s by more than average (Supplementary Table S1-3).

To assess how the numbers and positions of SAMRS components in an oligonucleotide affect T_m_, one to four standard bases near the 3′ of oligo were replaced by SAMRS (Supplementary Table S3). The T_m_s of the SAMRS:standard duplexes dropped by 0.1°C (**tgt**) to 6.8°C (**ggtc**), a range that depends on the number and the nature of SAMRS components. The greatest destabilization was seen with four SAMRS components, especially with **g**- and **c**-rich sequences. The positions where SAMRS components were introduced had little systematic effect (Supplementary Table S3). This is consistent with the T_m_ data collected in Supplementary Fig. S1 and Table S1. Again, introducing **g** causes the biggest decrease in T_m_, followed by introducing **c** and **a**; introducing **t** has the smallest change in T_m_.

To compare the T_m_ of SAMRS-containing duplexes with standard duplexes having one or two mismatches penultimate to the 3′-end, the G base close to the 3′-end of HIV-Std-Fp was replaced with either **g** or *A* (italics indicate mismatch) to give a **g**:C or *A*:C pair in duplex (Supplementary Table S3). The lowered melting temperature associated with the **g**:C (Supplementary Table S3, line 4, −1.2°C) was comparable to a single *A*:C mismatch (Supplementary Table S3, line 2, −1.9°C). The ΔT_m_ arising from replacing C penultimate to the 3′- of the HIV-Std-Rp with **c**, to give **c**:G (Supplementary Table S3, line 14, −0.6°C), was slightly smaller than a single *T*:G mismatch (Supplementary Table S3, line 9, −1.5°C).

### The PCR efficiency of primers containing four SAMRS components

Quantitative real-time PCR was used to amplify a short target (60 nt) with a common standard forward primer and a reverse primer containing four SAMRS components (Supplementary Table S2). All 256 reverse primers were designed to have approximately equal T_m_ values (∼60°C), their lengths were adjusted by adding up to three nucleobases at the 5′-end based on the heuristic parameters obtained above. In the template mixture, sites matching the SAMRS segment were synthesized to hold all four natural nucleotides. Thus, the SAMRS-containing reverse primer is seeking its perfectly complementary template that has 1/256 of the total template concentration.

Each 256 SAMRS-containing primer was then tested in PCRs with *Taq* DNA polymerase under two different concentrations of Mg^2+^ (1.5 or 5 mM). Results are reported in Supplementary Table S2, with the following outcomes summarized:

5. PCR efficiency depends on which SAMRS components are in the primers. The Ct’s of all 256 SAMRS-containing primers are from ∼16 to ∼24 cycles (at 5 mM, Supplementary Table S2-1). Thus:

5.1. **c**- and **g**-rich primers have lower amplification efficiency (**cccc**, **ccac**, **gcaa**, **ccca**, **cccg**, **ccgg**, **cggg**, **ccaa**, and **ccgc** have Ct values between 21 and 24 cycles).
5.2. **t**- and **a**-rich primers have higher amplification efficiency (i.e. **atgt**, **gagt**, **aagt**, **agta**, **aggt**, **aata**, and **atat** have Ct values between 16 and 17 cycles, Supplementary Table S2-1).
5.3. Primers have three or four consecutive **c’**s rank among the worst, followed by consecutive **g’**s, **t’**s, and **a’**s (Supplementary Table S2-2).

6. Lower Mg^2+^ concentration (1.5 mM) increased the sequence dependence of PCR performance.At 1.5 mM Mg^2+^, the worst SAMRS primers occasionally did not support amplification at all, even after 40 rounds of PCR, especially those containing more **g’**s and **c’**s. At 5 mM Mg^2+^, even these bad primers produced measurable (although not necessarily complete) amplification after 40 cycles; this agreed with the previous observation of increasing salt concentration appear to make **g**- and **c**-rich SAMRS sequences more like standard sequences in terms of T_m_.
7. To check the influence of the degeneracy of the template mixture on the relative PCR performance, a subset (ca. 40) of the primers was also subjected to PCR in the presence of their individually matching (nondegenerate) templates. No significant or systematic difference was observed, confirming the assumption that the degenerate template mixture used to evaluate the 256 SAMRS primers has a G:A:T:C ratio not materially different from 1:1:1:1, as delivered from IDT.

### The ability of SAMRS components to diminish the formation of primer dimers

To determine how few SAMRS components would help to diminish primer dimer formation, and where they are optimally placed, a “worst case” pair of primers were tested. These had six perfectly matched base pairs at their 3′-ends (Supplementary Fig. S2). Unsurprisingly, in the absence of target, the primer dimer was rapidly formed in PCR (Ct ∼8 cycles) with the standard primers (Std Primers) at all concentrations of Mg^2+^ (2, 3, and 4 mM). In contrast, the formation of primer dimer was delayed by ∼20–45 cycles (Table 1 and Fig. 2) with both forward and reverse primers containing different numbers and positions of SAMRS components.

In the presence of target (10^4^ copies), standard primers generated only primer dimer. However, SAMRS containing primers produced only amplicon or a mixture of amplicon and primer dimer. The ratio of PCR amplicon versus primer dimer depended on the types of SAMRS modifications (Table 2 and Fig. 3). This suggests a heuristic view of the need for placing SAMRS components near the 3′-end of the primer (1N or 2N) and two or more SAMRS modifications to achieve the best performance.

Summarizing these results:

8. Without target (Table 1), standard primers having complementary overlaps of six-base pairs at 3′-end gave primer dimer surpassing the threshold (Ct) in average after 7.9 cycles at 4, 3, or 2 mM of Mg^2+^. In contrast, primers with two SAMRS placed at positions 2 and 3 (2SAMRS-1N), three SAMRS at positions 2, 3, and 4 (3SAMRS-1N), and four SAMRS at positions 2, 3, 4, and 5 (4SAMRS-1N), delayed the formation of primer dimer in average after 21, 45, and 43 cycles, respectively (Fig. 2, left). This illustrates the value of SAMRS in preventing primer dimers.
9. Placing four SAMRS components in positions that are 1, 2, 3, and 4 nucleotides away from the 3′-end (4SAMRS-1N, 4SAMRS-2N, 4SAMRS-3N, and 4SAMRS-4N), also caused a delay in primer dimer formation (Fig. 2, right) in average after 43, 41, 22, and 20 cycles, respectively (Table 1). The Ct of primer dimer was further delayed by lowing Mg^2+^ concentration from 4 to 2 mM (Table 1).
10. With target (10^4^ copies), standard primers, still generated only primer dimer (T_m_ ≈ 81.5°C) after 7.6 cycles (Table 2 and Fig. 3). In contrast, primers containing three SAMRS (3SAMRS-1N) and four SAMRS (4SAMRS-1N and 4SAMRS-2N) all generated amplicons after ∼27 cycles (Table 2 and Fig. 3), while “preventing” formation of primer dimer, as shown by melting temperatures (Fig. 3, right, T_m_ ≈ 87.5°C). Primers with 2SAMRS-1N, 4SAMRS-3N, and 4SAMRS-4N all generated amplicons after ∼19 to ∼23 cycles (Table 2), melting at ∼87.5°C (Fig. 3, right).
11. We then measured the outcome if only one primer (here, the reverse primer, Supplementary Fig. S2) contains SAMRS components, while the forward primer is standard. Since SAMRS:standard pairs are stable, we expect this assay architecture to generate primer dimer. Here, the desired amplicon (Supplementary Fig. S2, left, T_m_ ≈ 88.5°C) was seen with some undesired primer dimer (T_m_ ≤ 81.5°C). The amount of the dimer depended on the numbers of the SAMRS components. Suppression of primer dimer is greatest with reverse primers containing the most SAMRS (Supplementary Fig. S2, left), respectively, 4, 3, 2, and 0 SAMRS components, the last (fully standard primer) showing essentially only primer dimer.
12. Primers were then examined where four SAMRS components were placed in positions that are 1, 2, 3, and 4 nucleotides further from the 3′-end of the reverse primer (1N, 2N, 3N, and 4N, Supplementary Fig. S2, right). Suppression of primer dimer was greatest in the primer with four SAMRS components closest to the 3′-end, 4SAMRS-1N and 4SAMRS-2N, respectively (Supplementary Fig. S2, right).

### SAMRS-containing primers support efficient PCR, suppress primer dimer, and improve SNPs discrimination

We then asked how SAMRS components can improve the ability of primers to amplify a challenging target. Here, the primer design is restricted for biological reasons to a specific region of the target. This allows for only limited optimization. As a target, we chose a conserved region of the reverse transcriptase gene in HIV1 (subtype B) that contains SNPs that confer resistance to the inexpensive HIV drugs [41]. This region is a challenging target because it is A/T rich.

To demonstrate the value of SAMRS to support PCR in a “least-worst case” design, we first tested the PCR efficiency of a common standard forward primer (HIV-Std-Fp) and a reverse primer modified with different numbers of SAMRS components. For standard primers, Ct values of the matched template G and no template control (NTC) are ∼30 and ∼36 cycles (Table 3). The Ct values of SAMRS-modified primers were decreased by 0, 0.3, 1.3, and 3.1 cycles with 1, 2, 3, and 4 SAMRS modifications which are proportional to the decreasing T_m_s of SAMRS primers. In addition, comparing 1SAMRS-T**c**-1N (Ct =  29.8) to 2SAMRS-**tc**-1N (Ct = 30.1); 2SAMRS-**a**T**c**-1N (Ct = 30.5) to 3SAMRS-**atc**-1N (Ct = 31.1); and 3SAMRS-**ga**T**c**-1N (Ct = 31.7) to 4SAMRS-**gatc**-1N (Ct = 32.9), the addition of **t** increases the Ct value by 0.3, 0.6 and 1.2, respectively (Table 3, match C:G).

These experiments also showed that primer dimers were suppressed by SAMRS modifications in a “no template control” reaction (Table 3, NTC). For standard primers, the Ct of primer dimer was delayed by 6.3 cycles than the PCR amplicon. For reverse primers modified with one up to four SAMRS components, the Ct of primer dimers were delayed by 7.2, 13.1, 24.1, and 25.2 cycles for 1, 2, 3, and 4 SAMRS modifications, respectively. With two SAMRS modifications, only one-third of experiments generated primer dimer; with three and four SAMRS (3SAMRS-**ga**T**c**-1N and 4SAMRS-**gatc**-1N), no primer dimer was observed, even after 60 cycles of PCR. Thus, the SAMRS modifications in the reverse primer can suppress primer dimer and support PCR amplification on challenging targets.

Interestingly, the amplification efficiency (30.1 cycles) of a standard reverse primer having one mismatch (Std-1Mis-C to *T*: template G) penultimate to the 3′-end of primer has the same efficiency as the reverse primer having two SAMRS components (2SAMRS-**tc**: template AG). Both amplification efficiencies were reduced by 0.3 cycles than the standard reverse primer (Table 3, match C:G). This may indicate that SAMRS components behave like semi-mismatches.

Often, researchers introduce a mismatch in the penultimate 3′-nucleotide into allele-specific primers to improve their ability to discriminate SNPs [42, 43]. Here, the discrimination principle assumes that a polymerase extends a primer with two mismatches at the penultimate and ultimate 3′ nucleotides (a “ragged end”) much less efficiently than it extends a primer with one mismatch and a perfectly matched primer. We reasoned that SAMRS modified primers might improve SNP discrimination as well.

As the results are shown in Table 3, the standard reverse primer (HIV-Std-Rp) can amplify both allele-G and allele-A templates (with G or A in the SNP site), and the Ct value of the allele-A template (mismatch C:A) was delayed by 2.3 cycles. Further, the standard reverse primer having one mismatch penultimate 3′ nucleotide (Std-1Mis-C to *T*) “generated only primer dimer,” both in the presence and absence of allele-A template (Table 3, mismatch C:A and NTC).

For reverse primers modified with different numbers of SAMRS, all SAMRS primers can amplify both allele-G and allele-A templates and offer a greater SNP discrimination. Quantitatively, comparing to the standard primer where the ΔCt of match vs. mismatch differs by 2.3 cycles, the ΔCt with SAMRS primers increased by 4.8, 5.6, 10.4, and 13.1 cycles, which are proportional to the number of SAMRS modifications (Table 3, mismatch C:A). Addition of one **t** to 2SAMRS-**a**T**c**-1N and 3SAMRS-**ga**T**c**-1N to give 3SAMRS-**atc**-1N and 4SAMRS-**gatc**-1N further increased SNP discrimination by ∼3–4 cycles (Table 3, mismatch C:A). These data provide a heuristic view of the ability of SAMRS primers to discriminate SNPs, “and also” has the benefit of suppressing primer dimer.

We then evaluated the performance of forward primers modified with different numbers of SAMRS. The amplification efficiencies were also decreased with increasing numbers of SAMRS modifications (Supplementary Table S4-1, match C:G). The formation of primer dimer was delayed by ∼14–17 cycles for primers containing three or four SAMRS components (Supplementary Table S4-1, NTC).

When both forward and reverse primers were modified with two, three, or four SAMRS components (2SAMRS-1N, 3SAMRS-1N, or 4SAMRS-1N), the Ct values for the matched template G were increased by ∼1, ∼3, or ∼10 cycles relative to the Ct of the standard primers (Supplementary Table S4-2, match C:G); the Ct values for the mismatched template A were increased by ∼5, ∼13, or 22 cycles, respectively (Supplementary Table S4-2, mismatch C:A). Strikingly, the primer dimer “remained undetectable” after 60 cycles of PCR (Supplementary Table S4-2, NTC).

### SAMRS modified primers with HiDi^™^ (high discrimination) DNA polymerase

We then asked whether results could be improved with the polymerases specifically developed to detect SNPs. One of these is the HiDi DNA polymerase (www.mypols.de), a thermostable variant of the large fragment of *KlenTaq* with increased mismatch selectivity for applications in allele- and methylation-specific amplification [44].

With the HiDi polymerase instead of *KlenTaq*, the Ct values of standard primers were the same for both polymerases with matched allele-G template (Table 4, match C:G); the Ct values of mismatched allele-A template were increased much more for HiDi polymerase (7.7 cycles, Table 4, mismatch C:A) than *KlenTaq* (2.3 cycles). For the reverse primers with different numbers of SAMRS, the Ct values of the matched template were increased by −0.9, 0.9, 3.4, and 6.7 cycles for 1, 2, 3, and 4 SAMRS modifications (Table 4, match C:G). However, the Ct values of the mismatched template were increased by much more, 26.1 and 21.8 cycles for one and two SAMRS modifications, and “no amplification (NA) at all” for 3 and 4 SAMRS modifications, even after 60 cycles (Table 4, mismatch C:A and NTC).

For a standard reverse primer having one mismatch (Std-1Mis C mispaired with *T*) penultimate to the 3′-end, the amplification efficiency of the allele-G template decreased by 4.1 cycles, almost the same level of decreasing as the 3SAMRS-**atc**-1N primer (Table 4, match C:G). It seems that the HiDi polymerase treats the T:G mismatch the same as a SAMRS:standard base pair (primer **atc**: template TAG) penultimate to the 3′-end primer. Indeed, the T_m_ of a duplex with one *T*:G mismatch (70.7°C) is close to the T_m_ of duplex with 3SAMRS:standard base pairs (71.0°C, **atc**:TAG, Supplementary Table S3). Further, 1/3 assays targeting on the mismatched allele-A template produced primer dimer (Table 4, mismatch C:A), and all assays without target generated primer dimer (Table 4, NTC). In contrast, all SAMRS-modified primers produced no primer dimers at all with the HiDi polymerase.

The same conclusions can be draw from the forward primer modified with SAMRS components using HiDi DNA polymerase as well as other commercially available polymerases, e.g. AmpliTaq God and SNPase HotStart DNA Polymerase (Bioron; data not shown).

We then assessed the generality of these observations with the goal of developing rules and recommendations for using SAMRS to improve PCR specificity and SNP detections. A set of primers and templates, published by IDT to benchmark the RNase H-dependent PCR (rhPCR) [11], were adopted to evaluate the performance of primers modified with SAMRS components.

Here a common standard forward primer (Common Std-Fp) was paired with standard reverse primers (Std-Rp-T-allele and Std-Rp-C-allele) and a set of reverse primers modified with SAMRS in different positions and numbers. Allele-specific PCR was performed by using *KlenTaq* and HiDi DNA polymerases with four synthetic templates where a single base was varied (A, G, C, or T).

For the T-allele reverse primers, the Ct values of the SAMRS modified reverse primers for the matched allele-A template are larger by 1.2–1.8 cycles than the Ct value of the standard reverse primer with *KlenTaq* (Table 5, match T:A). These observations agree with previous results: The amplification efficiency decreases as the numbers of SAMRS increasing and the position is closer to the 3′-end.

Again, SNP discrimination of the standard T-allele primer (Std-Rp-T-allele) showed ΔCt values ranging from 3 to 5.2 with an average of 4.4 for mismatched templates (allele-G, -C, and -T templates). SAMRS modified primers showed a higher level of SNP discrimination, with ΔCt values ranging from 6.2 to 17.2 with an average cycle delay of 9.9 with *KlenTaq* (Table 5, Mismatch T:G, T:C, and T:T).

SNP discrimination of SAMRS modified primers depends (as before) on the number and position of SAMRS components. The average ΔCt (12.4) of 4SAMRS-1N is larger than the average ΔCt of 3SAMRS-1N and 2SAMRS-1N (9.5 and 8.1, respectively). When placing four SAMRS away from the 3′-end, the average ΔCt of 4SAMRS-1N is also larger than the average ΔCt of 4SAMRS-2N, 4SAMRS-3N, and 4SAMRS-4N (12.0, 9.2, and 8.4, respectively). Consistent with the earlier results, SAMRS modified C-allele primers showed the same effects as the modified T-allele primers (Supplementary Table S5-1).

We then compared HiDi DNA polymerase with *KlenTaq* in parallel experiments. As before, HiDi polymerase gave slower PCR with SAMRS-containing primers than *KlenTaq*. The Ct values of the matched template were increased in average by ∼2.5 cycles with HiDi than with *KlenTaq* (Tables 5 and 6). This was the tradeoff for obtaining better SNP discrimination with HiDi. Thus, for the mismatched template G, the standard T-allele primer showed a ΔCt value of 9, in contrast, SAMRS modified T-allele primers showed ΔCt values ranging from ∼13 to NA (No Amplification). Specifically, 3SAMRS-1N, 4SAMRS-1N, and 4SAMRS-2N, had NA after 60 cycles; 2SAMRS-1N, 4SAMRS-3N, and 4SAMRS-4N, only 1/3 of assays showed amplification signals after ∼42 cycles (Table 6, mismatch T:G). For other mismatched templates (C and T), both standard T-allele primer and SAMRS modified T-allele primers showed essentially NA (Table 6, mismatch T:C and T:T).

For the C-allele primers, the ΔCt of the standard C-allele primer was 5.2 for the mismatched template A, and the ΔCt values of the SAMRS C-allele primers were ranged from 10.4 to 15.3 (Supplementary Table S5-2, mismatch C:A). For other mismatched templates (C and T), both standard C-allele primer and SAMRS modified C-allele primers showed essentially NA (Supplementary Table S5-2, mismatch C:C and C:T).

### Evaluation of SAMRS components and amplification sensitivity

As noted in the thermostability study, **t** was the best SAMRS component, “increasing” the strength of the pair to its natural complement A. In contrast, **g** was the worst (Supplementary Fig. S1 and Table S1). In addition, the PCR efficiency ranking shows that primers have consecutive **c’**s rank among the worst, followed by consecutive **g’**s, **t’**s, and **a’**s (Supplementary Table S2-2). Therefore, the PCR efficiency of SAMRS primers is not directly related to their thermostability. As we noticed that replacing T with **t** increases the T_m_ of SAMRS primer, however, decreases the amplification efficiency by 0.3–1.8 cycles (Tables 3 and 4).

To further understand how **t** and **c** affect the amplification efficiency of PCR with SAMRS primers, we compared primers with/without **t** and with/without three consecutive **c’**s. Here, the results are summarized in Supplementary Tables S6-1 and S6-2. Replacement of **t** by T was observed to modestly increase in PCR efficiency. As examples, upon **tgtg** going to T**g**T**g, ctgt** to **c**T**g**T, and **gctg** to **gc**T**g**, the appearance of product was faster by ∼0.3 cycles. Interestingly, replacement of **t** by T further delayed the formation of primer dimer by ∼4–9 cycles. This suggested that **t** might be less in preventing primer dimer formation than the rest of SAMRS components. Replacing one **c** by C (**accc** to **ac**C**c** or **ccct** to **c**C**c**T) to avoid three consecutive **c’**s was improving amplification efficiency by ∼0.5 cycles.

We further tested the efficiency and sensitivity of the SAMRS primers in which the rules for the optimal usage of SAMRS components were applied. Here, each forward and reverse primer contains two SAMRS components at positions 3 and 5 (**c**C**c**TG-3′ and **c**T**g**TT-3′). The PCR efficiencies (Ct’s) of SAMRS primers were compared to the standard primers (CCCTG-3′ and CTGTT-3′). Again, the Ct values of SAMRS primers were increased by ∼2–∼3 cycles at 10^4^, 10^3^, and 10^2^ copies of matched templates (Table 7, match T:A). However, in the absence of template (NTC), 3 out of 6 replicates gave primer dimer with standard primers after ∼39 cycles; only 1 out of 6 replicates gave a putative primer dimer with SAMRS primers after ∼43 cycles. For the mismatched templates, SAMRS primers increased SNP discrimination as before (Table 7, mismatch T:G).

An anonymous referee has suggested that 2-seleno-thymidine and/or 2-telluro-thymidine might be used as alternatives to **t**. Although the tellurium species is not yet reported, the selenium species is known, and has been used in crystallographic studies [45]. It forms a stable pair with adenosine. However, further development of this would need to understand the chemistry and enzymology of these species.

## Conclusion

These thermostability studies show that different SAMRS components contribute different levels of stability to SAMRS:standard pair. A duplex with single **t**:A pair is, in general, 0.8°C ± 0.4°C more stable than the same duplex with a T:A pair; a single **a**:T, **c**:G, or **g**:C pair is less stable than a corresponding standard pair (1.0°C ± 0.4°C, 3.0°C ± 0.5°C, or 4.5°C ± 0.5°C, respectively). For a typical four SAMRS-oligos (4SAMRS-1N, Supplementary Table S1) having a mixture of **g**, **c**, **a**, and **t**, in average, the T_m_ decrease by ∼4 to ∼5°C ± 2.7°C than the T_m_ of standard oligos. In addition, **g**- and **c**-rich SAMRS sequences decrease T_m_s by more than the average, **a**-rich sequences decrease less than the average, **taat** and **tata** sequences have little effect on ΔT_m_ (±0.3°C), and **t**-rich sequences (e.g. **tttt**), increase the T_m_s by 2.6°C. In addition, the T_m_s of oligos having greater numbers of SAMRS components are lower than the T_m_s of oligos having less SAMRS (Supplementary Table S3).

The ability of SAMRS primers to suppress primer dimer formation has been demonstrated by three case studies, which includes a challenging target and a “worst case” primer with six perfectly matched base pairs at 3′-ends (Tables 1–3, Figs 2 and 3).

For amplification efficiency, SAMRS modified primers, in general, delay the Ct values by ∼3 ± 2.5 cycles than the standard primers. This reflects a weaker binding of SAMRS primer to standard template. Thus, the amplification efficiency decreases as the numbers of SAMRS modifications increase and their positions are moved closer to the 3′-end (Tables 3–6). Further, amplification efficiencies are also influenced by the nature of the SAMRS components. In particular, primers having three or four consecutive **c’**s are the worst, followed by consecutive **g’**s, **t’**s, and **a’**s (Supplementary Table S2-2).

When SNP discrimination is desired, SAMRS primers can offer better SNP discrimination than standard primers in allele-specific PCR. Indeed, with HiDi DNA polymerase, SNP discrimination is outstanding. For a wide range of mismatches (T:G, T:C, T:T, C:C, and C:T), SAMRS-containing primers “generated no signal at all,” even after 60 cycles. This suggests that a combination of HiDi polymerase and SAMRS primers can be a choice for an extremely accurate SNP detection test (Table 6;Supplementary Table S5-2).

From the perspective of sensitivity, the value of SAMRS primers comes from their ability to generate extremely low background and essentially no primer dimer. Thus, SAMRS primers offer almost the same PCR sensitivity as the standard primers, even though they typically display a Ct delayed by ∼2–∼3 cycles (Table 7).

From these studies, certain general recommendations for the use of SAMRS primers can be summarized:

1. With SAMRS components, the primers should be lengthened, preferably to be over 20 nucleotides. This allows the T_m_s of SAMRS modified primers to be higher enough for efficient annealing to the target. This also increases the specificity footprint of the primers.
2. The number of SAMRS bases can be between one to four, with 1–3 SAMRS modifications being preferred. The ideal number of SAMRS modifications should be determined by the thermostability and sequence context of the 3′ end of the oligonucleotide.
3. SAMRS bases should be placed between the second up to eighth positions close to the 3′ end of the oligonucleotide, but not in the very first 3′ base. If the 3′-end of the oligonucleotide is T/A rich and the nucleotide at its 3′-end is a T or A, the penultimate nucleotide (Position 2) is recommended to be a standard base.
4. The SAMRS **t** base is the least effective in lowering primer dimer formation, making it preferable to substitute **a**, **g** or **c** rather than **t** when a choice is possible. In the case of 3′-T/A rich sequences, **t** can be used to stabilize the 3′-end of the SAMRS primers.
5. We recommend that consecutive **g’**s and **c’**s be avoided, as well as three or four consecutively identical SAMRS bases: **ggg**, **ccc**, **ttt**, **aaa**, **gggg**, **cccc**, **tttt**, and **aaaa**. Instead, we recommend separating **g’**s, **c’**s, **t’**s or **a’**s with standard nucleotides (G, C, A, T), e.g. **g**G**g**, **c**C**c**, **t**T**t**, and **a**A**a** in the SAMRS primers as the target sequence allows.
6. To improve the binding stability and specificity of **g** containing SAMRS primers, we recommend replacing G in the trimer (5′-NGN-3′) with **g** to give 5′-N**g**N-3′. The N’s are preferred to be C or G, based on the stability order of the nucleotide (N) on the 5′ side of **g** is: C > G > A > T; the stability order of the N on the 3′ side of **g** is: G > C ≈ A > T. Therefore, the preferred stable trimers are C**g**G, C**g**C, G**g**G, and G**g**C, the least stable trimers are A**g**T, T**g**A, and T**g**T.
7. To improve the amplification efficiency of **c**, the ethyl group on the 4-position nitrogen of **c** appears to create unfavorable interactions with the DNA polymerases. Accordingly, we recommend using N^4^-methyl-2′-deoxycytidine (^N4Me^C) as a new version of **c**, instead of N^4^-ethyl-2′-deoxycytidine (^N4Et^C). ^N4Me^C seems to be better accepted by polymerases [46, 47]. Interestingly, ^N4Me^C exists in some bacteria [48], which may explain its ability to serve as a polymerase substrate over ^N4Et^C. ^N4Me^C hybridizes specifically with natural G leading to a ^N4Me^C:G base pair whose stability is very close to that of the natural A:T base pair.

These general recommendations form a set of heuristics to design SAMRS modified primers for single-plexed PCR, as well as for multiplexed PCR. Again, the number of possible primer–primer interactions increases exponentially in multiplex PCR [13, 20]. With standard primers, the failure of multiplex PCR appears to be caused mainly by primer dimer resource wastage [22], even with low levels of multiplexing (∼10×). With SAMRS-modified primers, primer dimer formation is substantially suppressed. This should allow highly multiplexed PCR to be easily achieved without extensive optimization effort.

Indeed, two sets of 60 primers were designed for 30× multiplexed PCR by commercially available software. SAMRS components were introduced into these standard primers according to the rules developed here. Both sets of SAMRS primers were compared and benchmarked against standard primers. One set of SAMRS primers was tested in house and another set of SAMRS primers was tested by a third party. Both cases show that SAMRS primers perform much better than standard primers in 30× multiplexed PCR. These results will be published shortly.

The decision as to whether to use SAMRS in primers to support multiplex PCR depends on the specific objectives of an assay system. From the perspective of multiplex PCR, SAMRS primers significantly decrease the time and efforts needed to achieve a successful multiplex PCR. From the perspective of cost, standard primers are less expensive, although the cost differential will diminish if SAMRS-containing primers are widely adopted in multiplex PCR, allowing the scale of SAMRS phosphoramidite synthesis to increase and the price to decrease.

## Supplementary Material

bpaa004_Supplementary_Data
