# Supplementary material for: Eliminating primer dimers and improving SNP detection using self-avoiding molecular recognition systems
Source: Biol Methods Protoc. 2020 Feb 10;5(1):bpaa004. doi: 10.1093/biomethods/bpaa004 (PMC7200914; doi:10.1093/biomethods/bpaa004)
Supplement: bpaa004_Supplementary_Data [file bpaa004_supplementary_data.docx]

**Eliminating Primer Dimers and Improving SNP detection using Self-Avoiding Molecular Recognition Systems (SAMRS)**

Zunyi Yang ^1,2,^*, Jennifer T. Le ^1^, Daniel Hutter ^2^, Kevin M. Bradley ^1,2^, Benjamin R. Overton ^1^, Chris McLendon^1,2^, Steven A. Benner ^1,2,^*

* *To whom correspondence should be addressed.*

manuscripts@ffame.org; Tel: (+1) 386-418-8085;

Homepage: http://ffame.org and <http://firebirdbio.com>

^1^ Foundation for Applied Molecular Evolution (FfAME), 13709 Progress Blvd, Box 7, Alachua, FL 32615.

^2^ Firebird Biomolecular Sciences LLC, 13709 Progress Blvd, Box 17, Alachua, FL 32615, USA.

**SUPPLEMENTARY DATA**

Supplementary Data is available at Biology Methods and Protocols online.

**Supplementary Figure S1.**

Melting temperature difference (ΔT_m_) of X:Y

5'-ACCAAGC**X**ATCAAGT-3'

3'-TGGTTCG**Y**TAGTTCA-5'

**X** = A, **a**, T, **t**, G, **g,** C and **c.**

**Y** = T, **t**, A, **a**, C, **c**, G, and **g**.

ΔTm of X:Y (A:T as reference) under the conditions of 20 mM of Na cacodylate (pH 7.0), 100 mM of NaCl, 3.0 µM of each oligonucleotide.

**c** : G (0.25 ± 0.05 °C) > A : T (0 °C) > **t** : A (-0.1 ± 1.4 °C) > **a** : T (-2.0 ± 1 °C) > **g** : C (-2.2 ± 0.8 °C)


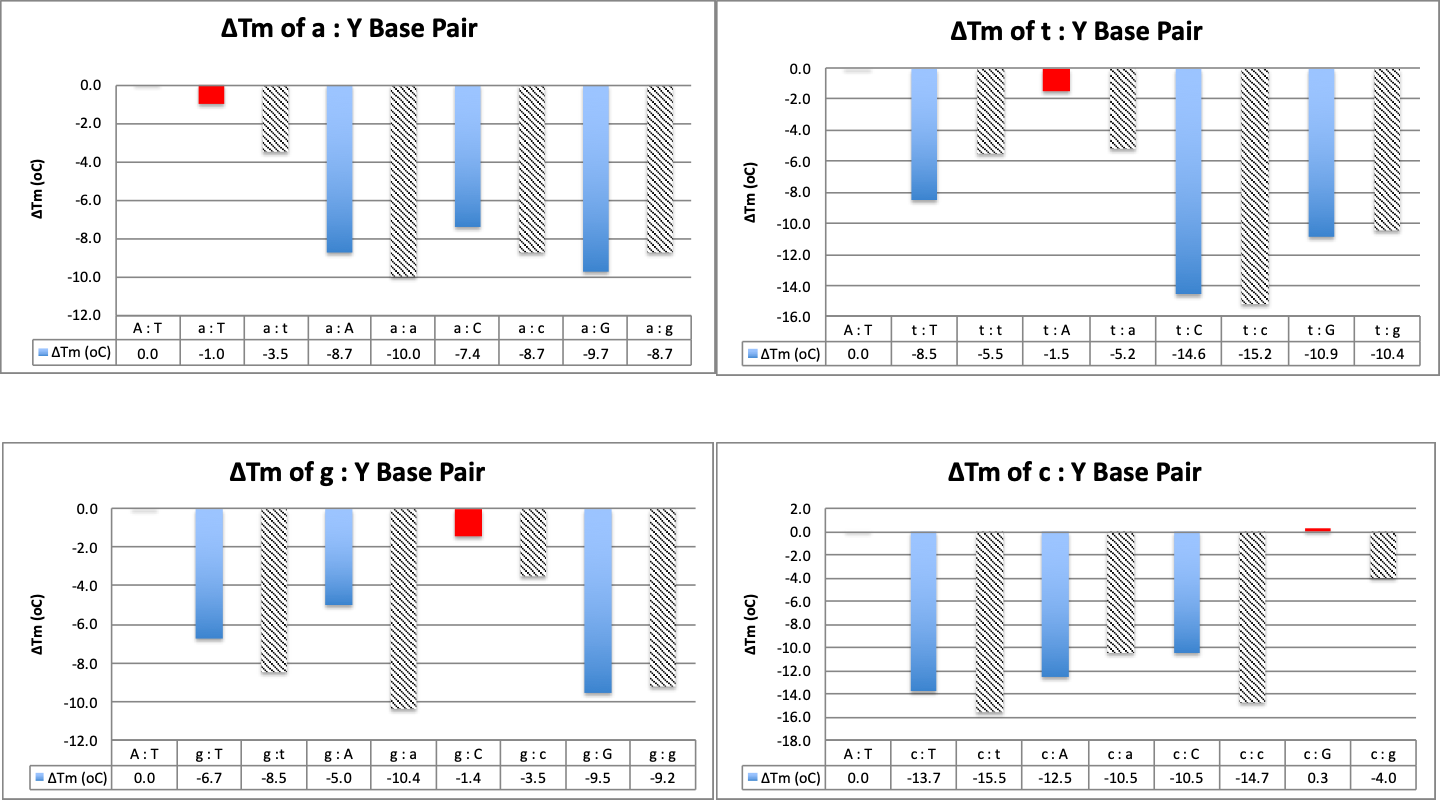


When literature data are combined, the following generalizations can be made about the stability of SAMRS:standard pairs relative to the standard pairs.

**g** : C (-4.5 ± 0.5 °C) < G:C; **g** : A (-9.0 ± 0.2 °C) < G:C; **g** : T (-11.3 ± 1.3 °C) < G:C;

**c** : G (-3.0 ± 0.5 °C) < C:G; **c** : A (-15.0 ± 2 °C) < C:G; **c** : **g** (-7.0 ± 0.4 °C) < C:G;

**a** : T (-1.0 ± 0.4 °C) < A:T; **a** : C (-7.2 ± 0.2 °C) < A:T; **a** : **t** (-3.5 ± 0.2 °C) < A:T;

**t** : A (+0.8 ± 0.4 °C) > T:A; **t** : G (-5.8 ± 0.2 °C) < T:A; **t** : **t** (-5.5 ± 0.2 °C) < T:A;

**Supplementary Table S1-1.**

Melting temperatures of standard oligos and SAMRS-containing oligos at 1.5 mM or 5 mM of MgCl_2_

Set 1 sequence: 5'-GAG CTG AGG TCA GTG T **n n n n** C-3'

Complementary sequences: 3'-CTC GAC TCC AGT CAC A **N N N N** G-5'

|  | Set 1 Duplex | 1.5 mM Mg++ | 1.5 mM Mg++ | 1.5 mM Mg++ | 5 mM Mg++ | 5 mM Mg++ | 5 mM Mg++ |
| --- | --- | --- | --- | --- | --- | --- | --- |
| Duplex # | SAMRS sequence | Average Tm of Standard Oligo | Average Tm of SAMRS Oligo | ∆Tm of SAMRS - Standard | Average Tm of Standard Oligo | Average Tm of SAMRS Oligo | ∆Tm of SAMRS - Standard |
| 1 | gcgc | 74.2 | 63.7 | -10.6 | 73.9 | 65.7 | -8.3 |
| 2 | gggg | 74.5 | 65.5 | -9.1 | 75.4 | 67.2 | -8.2 |
| 3 | gccg | 75.5 | 66.6 | -9.0 | 76.7 | 67.9 | -8.8 |
| 4 | cccc | 73.9 | 66.3 | -7.6 | 75.2 | 67.8 | -7.3 |
| 5 | tggg | 72.6 | 66.5 | -6.1 | 73.9 | 68.1 | -5.9 |
| 6 | cgct | 73.6 | 67.5 | -6.1 | 74.4 | 68.3 | -6.7 |
| 7 | ggaa | 70.4 | 65.0 | -5.5 | 71.8 | 66.4 | -5.4 |
| 8 | cgtc | 72.6 | 67.1 | -5.5 | 74.1 | 67.9 | -6.2 |
| 9 | gagt | 70.8 | 65.3 | -5.5 | 71.5 | 66.6 | -4.8 |
| 10 | ctgg | 72.6 | 67.4 | -5.3 | 74.0 | 68.2 | -5.8 |
| 11 | tgcc | 72.7 | 67.4 | -5.3 | 73.8 | 68.7 | -5.1 |
| 12 | gact | 71.0 | 65.8 | -5.2 | 72.3 | 67.6 | -4.7 |
| 13 | aagc | 70.1 | 64.9 | -5.2 | 71.6 | 66.4 | -5.2 |
| 14 | agga | 69.9 | 64.9 | -5.0 | 71.2 | 66.1 | -5.1 |
| 15 | cgat | 71.3 | 66.4 | -4.8 | 72.8 | 68.1 | -4.9 |
| 16 | atgg | 70.2 | 65.5 | -4.7 | 71.4 | 66.8 | -4.7 |
| 17 | acgt | 70.7 | 66.6 | -4.2 | 71.7 | 67.8 | -3.9 |
| 18 | catg | 70.9 | 66.9 | -4.0 | 72.3 | 68.5 | -3.8 |
| 19 | gtac | 70.0 | 66.3 | -3.7 | 71.2 | 67.5 | -3.7 |
| 20 | caca | 71.2 | 67.5 | -3.7 | 72.6 | 68.9 | -3.7 |
| 21 | atcc | 69.6 | 66.0 | -3.6 | 71.0 | 67.5 | -3.6 |
| 22 | gttc | 70.3 | 67.0 | -3.2 | 71.2 | 68.4 | -2.8 |
| 23 | tcca | 70.6 | 67.4 | -3.2 | 71.9 | 68.4 | -3.6 |
| 24 | caaa | 69.3 | 66.2 | -3.1 | 70.8 | 67.8 | -3.1 |
| 25 | tctg | 70.8 | 67.8 | -3.0 | 72.3 | 69.1 | -3.1 |
| 26 | cctt | 70.9 | 68.1 | -2.9 | 72.8 | 69.1 | -3.4 |
| 27 | agta | 68.2 | 65.4 | -2.8 | 69.4 | 66.5 | -2.9 |
| 28 | aatc | 67.8 | 65.4 | -2.4 | 69.1 | 67.0 | -2.1 |
| 29 | acat | 68.4 | 66.5 | -2.0 | 69.9 | 67.6 | -2.2 |
| 30 | aaaa | 66.8 | 65.1 | -1.7 | 68.1 | 66.2 | -1.9 |
| 31 | ttag | 68.9 | 67.5 | -1.4 | 70.3 | 69.0 | -1.3 |
| 32 | ttag | 68.5 | 67.4 | -1.1 | 69.9 | 68.8 | -1.0 |
| 33 | tatc | 67.7 | 66.9 | -0.8 | 69.3 | 68.4 | -0.8 |
| 34 | taat | 66.6 | 66.3 | -0.3 | 68.0 | 68.0 | 0.1 |
| 35 | tata | 66.5 | 66.8 | 0.3 | 68.0 | 68.3 | 0.2 |
| 36 | tttt | 67.3 | 69.9 | 2.6 | 68.8 | 71.4 | 2.6 |
| **Average Tm** |  | **70.5** | **66.5** | **-4.0** | **71.7** | **67.8** | **-3.9** |
| **Standard Deviation** |  | **2.3** | **1.2** | **2.7** | **2.2** | **1.1** | **2.5** |

**Supplementary Table S1-2.**

Melting temperatures of standard oligos and SAMRS-containing oligos at 1.5 mM and 5 mM of MgCl_2_

Set 2 sequence: 5'-GAG CTG AGG TCA GTG **N** **n a t n** **N**-3'

Complementary sequences: 3'-CTC GAC TCC AGT CAC **N** **N T A N** **N**-5'

|  | Set 2 Duplex | 1.5 mM Mg++ | 1.5 mM Mg++ | 1.5 mM Mg++ | 5 mM Mg++ | 5 mM Mg++ | 5 mM Mg++ |
| --- | --- | --- | --- | --- | --- | --- | --- |
| Duplex # | 3'-sequence | Average Tm of Standard Oligo | Average Tm of SAMRS Oligo | ∆Tm of SAMRS - Standard | Average Tm of Standard Oligo | Average Tm of SAMRS Oligo | ∆Tm of SAMRS - Standard |
| 1 | TgatgG | 70.5 | 65.1 | -5.4 | 71.0 | 67.1 | -3.9 |
| 2 | GgatgC | 71.8 | 66.7 | -5.1 | 73.1 | 68.5 | -4.6 |
| 3 | GgatgG | 71.5 | 66.8 | -4.7 | 72.1 | 68.6 | -3.5 |
| 4 | AgatcC | 69.3 | 64.8 | -4.5 | 71.1 | 66.8 | -4.2 |
| 5 | CgatcC | 72.6 | 68.2 | -4.4 | 74.0 | 69.9 | -4.1 |
| 6 | TgatcA | 69.2 | 65.0 | -4.2 | 70.8 | 66.7 | -4.2 |
| 7 | CgatgA | 71.7 | 67.7 | -4.0 | 73.5 | 69.9 | -3.2 |
| 8 | AaatgC | 68.3 | 64.4 | -3.9 | 70.2 | 65.9 | -4.3 |
| 9 | AaatgT | 67.6 | 64.4 | -3.3 | 69.3 | 65.9 | -3.3 |
| 10 | AaatcG | 68.2 | 65.0 | -3.3 | 70.1 | 66.5 | -3.6 |
| 11 | TcatcG | 70.1 | 66.9 | -3.2 | 71.8 | 68.6 | -3.2 |
| 12 | GcatgG | 72.5 | 69.3 | -3.1 | n/a | 70.5 | n/a |
| 13 | AcatcT | 69.3 | 66.3 | -3.0 | 71.3 | 68.3 | -3.0 |
| 14 | TgattC | 68.3 | 65.4 | -3.0 | 69.9 | 66.8 | -3.1 |
| 15 | CgattA | 70.8 | 67.9 | -2.9 | n/a | 70.0 | n/a |
| 16 | AgattT | 67.7 | 64.8 | -2.9 | 69.2 | 66.6 | -2.6 |
| 17 | GgataA | 69.1 | 66.4 | -2.7 | 71.3 | 68.3 | -3.0 |
| 18 | TcatcT | 69.6 | 67.2 | -2.5 | 71.1 | 68.8 | -2.4 |
| 19 | AgattG | 68.2 | 65.7 | -2.5 | 69.8 | 67.2 | -2.6 |
| 20 | GaatgT | 68.9 | 66.4 | -2.5 | 70.7 | 68.6 | -2.1 |
| 21 | CcatcC | 72.1 | 69.9 | -2.3 | n/a | 70.4 | n/a |
| 22 | CcatcA | 71.9 | 69.7 | -2.2 | 73.8 | 71.4 | -3.8 |
| 23 | TcataT | 68.5 | 66.7 | -1.8 | 70.3 | 68.2 | -2.1 |
| 24 | GcataA | 70.7 | 69.0 | -1.8 | 72.8 | 71.0 | -1.8 |
| 25 | AcataG | 68.6 | 66.8 | -1.8 | 70.6 | 68.4 | -2.2 |
| 26 | TaatcT | 66.8 | 65.1 | -1.7 | 68.5 | 66.8 | -1.6 |
| 27 | CtatgC | 70.1 | 68.7 | -1.4 | 71.4 | 70.7 | -0.7 |
| 28 | GaataC | 68.0 | 66.6 | -1.4 | 69.8 | 68.4 | -1.4 |
| 29 | AcattG | 68.9 | 67.6 | -1.3 | 70.6 | 69.1 | -1.6 |
| 30 | GcattA | 70.8 | 69.7 | -1.2 | 73.0 | 70.8 | -2.2 |
| 31 | TaataC | 65.9 | 64.7 | -1.2 | 67.7 | 66.5 | -1.2 |
| 32 | CcattC | 71.2 | 70.2 | -1.0 | 73.3 | 71.3 | -2.5 |
| 33 | TtatcG | 67.8 | 66.9 | -1.0 | 69.5 | 68.5 | -1.0 |
| 34 | GaataT | 67.4 | 66.4 | -1.0 | 69.0 | 68.4 | -0.8 |
| 35 | GtatgA | 69.0 | 68.2 | -0.8 | 70.6 | 70.3 | -0.4 |
| 36 | AtatgT | 67.0 | 66.2 | -0.7 | 68.5 | 67.9 | -0.5 |
| 37 | CaataA | 68.7 | 68.2 | -0.6 | 71.0 | 70.0 | -1.0 |
| 38 | TaataT | 65.6 | 65.1 | -0.6 | 67.3 | 66.6 | -0.7 |
| 39 | CaattT | 68.8 | 68.2 | -0.5 | 70.4 | 70.0 | -0.4 |
| 40 | CaattG | 69.3 | 68.7 | -0.5 | 71.1 | 70.6 | -0.5 |
| 41 | CtatgA | 69.1 | 68.9 | -0.2 | 70.7 | 70.5 | -0.2 |
| 42 | CtatcA | 69.1 | 69.0 | 0.0 | 70.9 | 70.8 | 0.0 |
| 43 | GtataG | 68.0 | 68.4 | 0.4 | 69.8 | 70.0 | 0.2 |
| 44 | AtataG | 65.9 | 66.2 | 0.4 | 67.4 | 68.1 | 0.7 |
| 45 | GtataC | 67.7 | 68.3 | 0.5 | 69.6 | 69.8 | 0.2 |
| 46 | TtattT | 66.2 | 67.2 | 1.0 | 68.1 | 68.8 | 0.7 |
| 47 | TtattA | 65.7 | 66.9 | 1.2 | 67.6 | 68.4 | 0.8 |
| 48 | AtattC | 65.9 | 67.2 | 1.3 | 67.5 | 68.9 | 1.4 |
| **Average Tm** |  | **69.0** | **67.1** | **-1.9** | **70.5** | **68.8** | **-1.9** |
| **Standard Deviation** |  | **1.9** | **1.6** | **1.7** | **1.7** | **1.6** | **1.6** |

**Supplementary Table S1-3.**

Melting temperatures of standard oligos and SAMRS-containing oligos at 1.5 mM of MgCl_2_

Set 3 sequence: 5'-GCT CGA ATT GCA CCC T **n n n n** C-3`

Complementary sequences: 3'-CGA GCT TAA CGT GGG A **N** **N N** **N** G-5'

|  | Set 3 Duplex | 1.5 mM Mg++ | 1.5 mM Mg++ | 1.5 mM Mg++ |
| --- | --- | --- | --- | --- |
| Duplex # | SAMRS sequence | Average Tm of Standard Primer | Average Tm of SAMRS Primer | ∆Tm of SAMRS - Standard |
| 1 | cttt | 63.0 | 63.7 | 0.7 |
| 2 | aatt | 64.4 | 64.1 | -0.3 |
| 3 | gttt | 64.0 | 63.0 | -1.0 |
| 4 | taca | 64.3 | 63.3 | -1.0 |
| 5 | tcta | 63.6 | 61.5 | -2.1 |
| 6 | attg | 64.2 | 62.1 | -2.1 |
| 7 | aaat | 65.9 | 63.8 | -2.1 |
| 8 | atag | 65.2 | 62.9 | -2.3 |
| 9 | ctag | 67.3 | 64.8 | -2.5 |
| 10 | cata | 65.9 | 63.0 | -2.9 |
| 11 | tctt | 63.2 | 60.0 | -3.2 |
| 12 | taga | 66.0 | 62.5 | -3.5 |
| 13 | ctcc | 67.6 | 63.8 | -3.8 |
| 14 | cgta | 67.9 | 64.1 | -3.8 |
| 15 | tcct | 66.6 | 62.7 | -3.9 |
| 16 | ttcg | 66.5 | 62.6 | -3.9 |
| 17 | taac | 64.2 | 60.3 | -3.9 |
| 18 | ccat | 67.9 | 63.9 | -4.0 |
| 19 | agag | 68.3 | 64.1 | -4.2 |
| 20 | tgac | 66.6 | 62.3 | -4.3 |
| 21 | tgtc | 66.5 | 62.2 | -4.3 |
| 22 | gtgt | 68.3 | 64.0 | -4.3 |
| 23 | atgc | 67.8 | 63.5 | -4.3 |
| 24 | acct | 67.7 | 63.3 | -4.4 |
| 25 | tgct | 67.7 | 63.1 | -4.6 |
| 26 | gatc | 67.9 | 63.2 | -4.7 |
| 27 | caag | 67.9 | 63.0 | -4.9 |
| 28 | aact | 65.5 | 60.2 | -5.3 |
| 29 | acac | 66.5 | 61.1 | -5.4 |
| 30 | gttg | 66.5 | 60.9 | -5.6 |
| 31 | gcaa | 67.7 | 61.7 | -6.0 |
| 32 | acca | 68.4 | 62.4 | -6.0 |
| 33 | ccca | 68.8 | 62.2 | -6.6 |
| 34 | cggt | 69.8 | 63.0 | -6.8 |
| 35 | tagc | 66.4 | 59.6 | -6.8 |
| 36 | gtcc | 67.7 | 60.6 | -7.1 |
| 37 | aggc | 70.0 | 62.8 | -7.2 |
| 38 | aagg | 67.0 | 59.7 | -7.3 |
| 39 | cgtg | 69.5 | 62.1 | -7.4 |
| 40 | cgga | 69.3 | 61.8 | -7.5 |
| 41 | gcga | 71.4 | 63.4 | -8.0 |
| 42 | gcca | 70.1 | 61.3 | -8.8 |
| 43 | ggga | 70.1 | 60.9 | -9.2 |
| 44 | gatg | 69.0 | 59.1 | -9.9 |
| 45 | gaac | 68.1 | 57.3 | -10.8 |
| 46 | cggc | 69.5 | 57.8 | -11.7 |
| 47 | tcgg | 67.5 | 55.7 | -11.8 |
| 48 | gcgg | 70.4 | 57.7 | -12.7 |
| **Average Tm** |  | **67.2** | **61.9** | **-5.3** |
| **Standard Deviation** |  | **2.0** | **2.0** | **3.0** |

**Supplementary Table S2.**

Evaluation of amplification efficiency of 256 SAMRS-containing primers by Real time PCR

5'-TAC GGC TAT GGA CAT CAC-3'

5'-TAC GGC TAT GGA CAT CAC ATTCAGCGCAAATCAGGTAAG G **N N N N** A GGG TGC AAT TCG AGC-3'

3`-C **n n n n** T CCC ACG TTA AGC(TCG)-5'

**Supplementary Table S2-1.**

PCR efficiency (Ct) ranks of 256 SAMRS-modified reverse primers at 5 mM of Mg^2+^.

| **Rank by Ct** | **SAMRS sequence** | **Average Ct** | **Rank by Ct** | **SAMRS sequence** | **Average Ct** | **Rank by Ct** | **SAMRS sequence** | **Average Ct** | **Rank by Ct** | **SAMRS sequence** | **Average Ct** |
| --- | --- | --- | --- | --- | --- | --- | --- | --- | --- | --- | --- |
| **1** | atgt | 16.4 | **65** | tatc | 18.1 | **129** | actc | 18.7 | **193** | tgcc | 19.5 |
| **2** | gagt | 16.6 | **66** | ggtt | 18.1 | **130** | catt | 18.7 | **194** | tacg | 19.5 |
| **3** | aagt | 16.7 | **67** | attt | 18.1 | **131** | gacg | 18.7 | **195** | accc | 19.5 |
| **4** | agta | 16.7 | **68** | cagt | 18.1 | **132** | tttc | 18.7 | **196** | ccat | 19.6 |
| **5** | aggt | 16.8 | **69** | taga | 18.1 | **133** | caat | 18.8 | **197** | accg | 19.6 |
| **6** | aata | 16.9 | **70** | gaac | 18.1 | **134** | acga | 18.8 | **198** | cttg | 19.6 |
| **7** | atat | 17.0 | **71** | gcta | 18.1 | **135** | gcga | 18.8 | **199** | tggc | 19.6 |
| **8** | atga | 17.0 | **72** | tatg | 18.1 | **136** | tgca | 18.8 | **200** | tctg | 19.7 |
| **9** | gaat | 17.2 | **73** | aaca | 18.1 | **137** | gtca | 18.8 | **201** | tgcg | 19.7 |
| **10** | aaat | 17.2 | **74** | gggt | 18.1 | **138** | gtgg | 18.8 | **202** | ggca | 19.7 |
| **11** | gatt | 17.2 | **75** | atca | 18.2 | **139** | gttg | 18.8 | **203** | ctcc | 19.8 |
| **12** | tact | 17.3 | **76** | ttgt | 18.2 | **140** | ctag | 18.8 | **204** | cctg | 19.8 |
| **13** | agtt | 17.3 | **77** | gtta | 18.2 | **141** | gttc | 18.8 | **205** | gctc | 19.8 |
| **14** | tagt | 17.3 | **78** | aacg | 18.2 | **142** | acac | 18.8 | **206** | tctc | 19.8 |
| **15** | gatc | 17.4 | **79** | agcg | 18.2 | **143** | gctg | 18.9 | **207** | gcca | 19.8 |
| **16** | agag | 17.4 | **80** | acct | 18.2 | **144** | tcgt | 18.9 | **208** | cctc | 19.8 |
| **17** | agat | 17.4 | **81** | gtac | 18.2 | **145** | ggag | 18.9 | **209** | gcag | 19.8 |
| **18** | gtgt | 17.4 | **82** | taaa | 18.2 | **146** | gcgt | 18.9 | **210** | tcac | 19.8 |
| **19** | agtc | 17.4 | **83** | attc | 18.2 | **147** | ttag | 18.9 | **211** | cttc | 19.8 |
| **20** | atta | 17.4 | **84** | gtga | 18.2 | **148** | ctga | 18.9 | **212** | gtcc | 19.8 |
| **21** | gatg | 17.4 | **85** | attg | 18.2 | **149** | ccta | 18.9 | **213** | gtcg | 19.8 |
| **22** | gaga | 17.4 | **86** | tgtc | 18.2 | **150** | tcat | 18.9 | **214** | ttca | 19.9 |
| **23** | atgc | 17.5 | **87** | acat | 18.2 | **151** | cgat | 19.0 | **215** | cacc | 20.0 |
| **24** | aatt | 17.5 | **88** | atag | 18.3 | **152** | acgc | 19.0 | **216** | cgca | 20.0 |
| **25** | gagc | 17.5 | **89** | ggtg | 18.3 | **153** | ctaa | 19.0 | **217** | tgaa | 20.1 |
| **26** | tata | 17.5 | **90** | agaa | 18.3 | **154** | caga | 19.0 | **218** | ttcg | 20.2 |
| **27** | ataa | 17.6 | **91** | agca | 18.3 | **155** | tcta | 19.0 | **219** | ttaa | 20.2 |
| **28** | tgta | 17.6 | **92** | ctct | 18.3 | **156** | caaa | 19.0 | **220** | ttcc | 20.2 |
| **29** | aaga | 17.6 | **93** | agac | 18.3 | **157** | aaaa | 19.0 | **221** | gcgc | 20.2 |
| **30** | actt | 17.6 | **94** | gtgc | 18.3 | **158** | ctta | 19.0 | **222** | ccga | 20.3 |
| **31** | agga | 17.6 | **95** | tgag | 18.3 | **159** | catc | 19.1 | **223** | cgac | 20.3 |
| **32** | agtg | 17.6 | **96** | tgtg | 18.3 | **160** | cagc | 19.1 | **224** | ggcg | 20.3 |
| **33** | aatg | 17.7 | **97** | atcc | 18.3 | **161** | catg | 19.1 | **225** | ccgt | 20.3 |
| **34** | gact | 17.7 | **98** | ggac | 18.4 | **162** | cgtc | 19.1 | **226** | tggg | 20.4 |
| **35** | aatc | 17.7 | **99** | tacc | 18.4 | **163** | tgac | 19.1 | **227** | gcac | 20.4 |
| **36** | agct | 17.7 | **100** | aagg | 18.4 | **164** | caag | 19.1 | **228** | ccct | 20.4 |
| **37** | aagc | 17.8 | **101** | gtaa | 18.4 | **165** | ttgc | 19.1 | **229** | cgcc | 20.4 |
| **38** | gtct | 17.8 | **102** | aggg | 18.4 | **166** | ctgc | 19.1 | **230** | ggcc | 20.4 |
| **39** | tggt | 17.8 | **103** | ggtc | 18.4 | **167** | gcct | 19.1 | **231** | tcgc | 20.4 |
| **40** | aact | 17.8 | **104** | gacc | 18.4 | **168** | ggga | 19.1 | **232** | tccc | 20.5 |
| **41** | gtat | 17.8 | **105** | taac | 18.4 | **169** | acgg | 19.1 | **233** | tcga | 20.5 |
| **42** | ggta | 17.8 | **106** | atcg | 18.4 | **170** | caac | 19.2 | **234** | cggc | 20.6 |
| **43** | taat | 17.8 | **107** | acag | 18.4 | **171** | ctac | 19.2 | **235** | cacg | 20.6 |
| **44** | tatt | 17.8 | **108** | gcat | 18.5 | **172** | ctca | 19.2 | **236** | cgcg | 20.6 |
| **45** | acgt | 17.8 | **109** | cata | 18.5 | **173** | cgtg | 19.2 | **237** | gggc | 20.6 |
| **46** | tgtt | 17.9 | **110** | cgta | 18.5 | **174** | ttac | 19.2 | **238** | gcgg | 20.7 |
| **47** | gata | 17.9 | **111** | ttat | 18.5 | **175** | caca | 19.2 | **239** | gccc | 20.8 |
| **48** | aggc | 17.9 | **112** | tgct | 18.6 | **176** | gaaa | 19.3 | **240** | tccg | 20.8 |
| **49** | atct | 17.9 | **113** | gaag | 18.6 | **177** | tcct | 19.3 | **241** | ccag | 20.9 |
| **50** | atac | 17.9 | **114** | gctt | 18.6 | **178** | tcag | 19.3 | **242** | tcgg | 20.9 |
| **51** | aacc | 17.9 | **115** | tagg | 18.6 | **179** | cgct | 19.3 | **243** | gccg | 20.9 |
| **52** | acta | 17.9 | **116** | tttt | 18.6 | **180** | ttga | 19.3 | **244** | ctcg | 20.9 |
| **53** | tgat | 17.9 | **117** | aaag | 18.6 | **181** | cctt | 19.3 | **245** | cgaa | 21.0 |
| **54** | actg | 17.9 | **118** | cggt | 18.6 | **182** | cgag | 19.3 | **246** | ggaa | 21.1 |
| **55** | gtag | 17.9 | **119** | tttg | 18.6 | **183** | tctt | 19.3 | **247** | tcaa | 21.1 |
| **56** | agcc | 18.0 | **120** | ggct | 18.6 | **184** | cgga | 19.4 | **248** | ccgc | 21.1 |
| **57** | cact | 18.0 | **121** | acaa | 18.7 | **185** | ttta | 19.4 | **249** | ccaa | 21.1 |
| **58** | gaca | 18.0 | **122** | tgga | 18.7 | **186** | cagg | 19.4 | **250** | cggg | 21.3 |
| **59** | taag | 18.0 | **123** | taca | 18.7 | **187** | tcca | 19.4 | **251** | ccgg | 21.5 |
| **60** | atgg | 18.0 | **124** | ctat | 18.7 | **188** | ttct | 19.4 | **252** | cccg | 22.0 |
| **61** | gagg | 18.0 | **125** | ggat | 18.7 | **189** | ttgg | 19.5 | **253** | ccca | 22.1 |
| **62** | ctgt | 18.0 | **126** | acca | 18.7 | **190** | cttt | 19.5 | **254** | gcaa | 22.8 |
| **63** | aaac | 18.0 | **127** | cgtt | 18.7 | **191** | ctgg | 19.5 | **255** | ccac | 22.8 |
| **64** | tagc | 18.0 | **128** | gttt | 18.7 | **192** | gggg | 19.5 | **256** | cccc | 24.0 |

**Supplementary Table S2-2.**

Ct rank of primers containing consecutive SAMRS components.

Ct rank of 4 consecutive SAMRS components:

| **4SAMRS** | **Rank by Ct** |
| --- | --- |
| tttt | 116 |
| aaaa | 157 |
| gggg | 192 |
| cccc | 256 |

Ct rank of 3 consecutive SAMRS components:

| **aaa** | **Rank by Ct** | **ttt** | **Rank by Ct** | **ggg** | **Rank by Ct** | **ccc** | **Rank by Ct** |
| --- | --- | --- | --- | --- | --- | --- | --- |
| aaat | 10 | attt | 67 | gggt | 74 | accc | 195 |
| aaac | 63 | tttt | 116 | aggg | 102 | ccct | 228 |
| taaa | 82 | tttg | 119 | ggga | 168 | tccc | 232 |
| aaag | 117 | gttt | 128 | gggg | 192 | gccc | 239 |
| caaa | 156 | tttc | 132 | tggg | 226 | cccg | 252 |
| aaaa | 157 | ttta | 185 | gggc | 237 | ccca | 253 |
| gaaa | 176 | cttt | 190 | cggg | 250 | cccc | 256 |
| **Average Ct** | **109** |  | **134** |  | **178** |  | **236** |

Ct rank of 2 consecutive SAMRS components:

| **aa** | **Rank by Ct** | **tt** | **Rank by Ct** | **gg** | **Rank by Ct** | **cc** | **Rank by Ct** |
| --- | --- | --- | --- | --- | --- | --- | --- |
| aagt | 3 | gatt | 11 | aggt | 5 | aacc | 51 |
| aata | 6 | agtt | 13 | agga | 31 | agcc | 56 |
| gaat | 9 | atta | 20 | tggt | 39 | acct | 80 |
| aaat | 10 | aatt | 24 | ggta | 42 | atcc | 97 |
| aatt | 24 | actt | 30 | aggc | 48 | tacc | 99 |
| ataa | 27 | tatt | 44 | atgg | 60 | gacc | 104 |
| aaga | 29 | tgtt | 46 | gagg | 61 | acca | 126 |
| aatg | 33 | ggtt | 66 | ggtt | 66 | ccta | 149 |
| aatc | 35 | attt | 67 | gggt | 74 | gcct | 167 |
| aagc | 37 | ttgt | 76 | ggtg | 89 | tcct | 177 |
| aact | 40 | gtta | 77 | ggac | 98 | cctt | 181 |
| taat | 43 | attc | 83 | aagg | 100 | tcca | 187 |
| aacc | 51 | attg | 85 | aggg | 102 | tgcc | 193 |
| taag | 59 | ttat | 111 | ggtc | 103 | accc | 195 |
| aaac | 63 | gctt | 114 | tagg | 115 | ccat | 196 |
| gaac | 70 | tttt | 116 | cggt | 118 | accg | 197 |
| aaca | 73 | tttg | 119 | ggct | 120 | ctcc | 203 |
| aacg | 78 | cgtt | 127 | tgga | 122 | cctg | 204 |
| taaa | 82 | gttt | 128 | ggat | 125 | gcca | 207 |
| agaa | 90 | catt | 130 | gtgg | 138 | cctc | 208 |
| aagg | 100 | tttc | 132 | ggag | 145 | gtcc | 212 |
| gtaa | 101 | gttg | 139 | ggga | 168 | cacc | 215 |
| taac | 105 | gttc | 141 | acgg | 169 | ttcc | 220 |
| gaag | 113 | ttag | 147 | cgga | 184 | ccga | 222 |
| aaag | 117 | ctta | 158 | cagg | 186 | ccgt | 225 |
| acaa | 121 | ttgc | 165 | ttgg | 189 | ccct | 228 |
| caat | 133 | ttac | 174 | ctgg | 191 | cgcc | 229 |
| ctaa | 153 | ttga | 180 | gggg | 192 | ggcc | 230 |
| caaa | 156 | cctt | 181 | tggc | 199 | tccc | 232 |
| aaaa | 157 | tctt | 183 | ggca | 202 | gccc | 239 |
| caag | 164 | ttta | 185 | ggcg | 224 | tccg | 240 |
| caac | 170 | ttct | 188 | tggg | 226 | ccag | 241 |
| gaaa | 176 | ttgg | 189 | ggcc | 230 | gccg | 243 |
| tgaa | 217 | cttt | 190 | cggc | 234 | ccgc | 248 |
| ttaa | 219 | cttg | 198 | gggc | 237 | ccaa | 249 |
| cgaa | 245 | cttc | 211 | gcgg | 238 | ccgg | 251 |
| ggaa | 246 | ttca | 214 | tcgg | 242 | cccg | 252 |
| tcaa | 247 | ttcg | 218 | ggaa | 246 | ccca | 253 |
| ccaa | 249 | ttaa | 219 | cggg | 250 | ccac | 255 |
| gcaa | 254 | ttcc | 220 | ccgg | 251 | cccc | 256 |
| **Average Ct** | **108** |  | **128** |  | **146** |  | **195** |

**The Ct of four SAMRS-containing primer (rank from 1 to 48)**

**The Ct of four SAMRS-containing primer (rank from 49 to 96)**

**The Ct of four SAMRS-containing primer (rank from 97 to 144)**

**The Ct of four SAMRS-containing primer (rank from 193 to 224)**

**The Ct of four SAMRS-containing primer (rank from 225 to 256)**

**Supplementary Table S3.**

Melting temperatures of standard oligos and SAMRS-containing oligos were tested in 1x *KlenTaq* Buffer (50 mM Tris (pH 8.3), 0.25 µg/µL BSA, 1x LC Green, and 3 mM Mg^2+^).

The standard complementary oligos of each SAMRS oligo (1µM) were not shown.

| **Name** | **Sequence (5'- to 3')** | **T_m_ (^o^C)** | **ΔT_m_ (^o^C)** |
| --- | --- | --- | --- |
| HIV-Std-Fp-30 | 5’-TCCAAAGTAGCATGACAAAAATCTTAGAGC-3' | 70.8 | 0.0 |
| Std-Fp-1Mis-*A* | 5’-TCCAAAGTAGCATGACAAAAATCTTAGA*A*C-3' | 68.9 | -1.9 |
| 2SAMRS-gAg-1N | 5’-TCCAAAGTAGCATGACAAAAATCTTAgAgC-3' | 68.2 | -2.6 |
| 1SAMRS-g-1N | 5’-TCCAAAGTAGCATGACAAAAATCTTAGAgC-3' | 69.6 | -1.2 |
| 2SAMRS-ag-1N | 5’-TCCAAAGTAGCATGACAAAAATCTTAGagC-3' | 69.5 | -1.3 |
| 3SAMRS-gag-1N | 5’-TCCAAAGTAGCATGACAAAAATCTTAgagC-3' | 68.2 | -2.6 |
| 4SAMRS-agag-1N | 5’-TCCAAAGTAGCATGACAAAAATCTTagagC-3' | 67.9 | -2.9 |
| **Name** | **Sequence (5'- to 3')** | **T_m_ (^o^C)** | **ΔT_m_ (^o^C)** |
| HIV-Std-Rp-27 | 5'-ATGCTGCCCTATTTCTAAGTCAGATCC-3' | 72.2 | 0.0 |
| Std-Rp-1Mis-*T* | 5'-ATGCTGCCCTATTTCTAAGTCAGAT*T*C-3' | 70.7 | -1.5 |
| Std-Rp-2Mis-*CT* | 5'-ATGCTGCCCTATTTCTAAGTCAGA*CT*C-3' | 70.4 | -1.8 |
| 2SAMRS-gATc-1N | 5'-ATGCTGCCCTATTTCTAAGTCAgATcC-3' | 69.4 | -2.8 |
| 2SAMRS-aTc-1N | 5'-ATGCTGCCCTATTTCTAAGTCAGaTcC-3' | 70.9 | -1.3 |
| 3SAMRS-gaTc-1N | 5`-ATGCTGCCCTATTTCTAAGTCAgaTcC-3` | 68.8 | -3.4 |
| 1SAMRS-Tc-1N | 5`-ATGCTGCCCTATTTCTAAGTCAGATcC-3` | 71.6 | -0.6 |
| 2SAMRS-tc-1N | 5`-ATGCTGCCCTATTTCTAAGTCAGAtcC-3` | 71.8 | -0.4 |
| 3SAMRS-atc-1N | 5`-ATGCTGCCCTATTTCTAAGTCAGatcC-3` | 71.0 | -1.2 |
| 4SAMRS-gatc-1N | 5`-ATGCTGCCCTATTTCTAAGTCAgatcC-3` | 68.8 | -3.4 |

| **Name** | **Sequence (5'- to 3')** | **T_m_ (^o^C)** | **ΔT_m_ (^o^C)** |
| --- | --- | --- | --- |
| Std-Fp-25 | 5’-TATCTGCGTGCCCTGTCTCTGGAGG-3’ | 76.7 | 0.0 |
| 3SAMRS-1N | 5’-TATCTGCGTGCCCTGTCTCTGgagG-3’ | 75.3 | -1.3 |
| 4SAMRS-1N | 5’-TATCTGCGTGCCCTGTCTCTggagG-3’ | 72.7 | -4.0 |
| 4SAMRS-2N | 5’-TATCTGCGTGCCCTGTCTCtggaGG-3’ | 74.0 | -2.6 |
| 4SAMRS-3N | 5’-TATCTGCGTGCCCTGTCTctggAGG-3’ | 72.7 | -3.9 |
| 4SAMRS-4N | 5’-TATCTGCGTGCCCTGTCtctgGAGG-3’ | 74.2 | -2.5 |
|  |  |  |  |
| Std-Rp-25 | 5’-CCAATGCCAACATCTACCTCCAGAG-3’ | 73.1 | 0.0 |
| 2SAMRS-1N | 5’-CCAATGCCAACATCTACCTCCAgaG-3’ | 71.9 | -1.3 |
| 3SAMRS-1N | 5’-CCAATGCCAACATCTACCTCCagaG-3’ | 71.9 | -1.3 |
| 4SAMRS-1N | 5’-CCAATGCCAACATCTACCTCcagaG-3’ | 70.5 | -2.7 |
| 4SAMRS-2N | 5’-CCAATGCCAACATCTACCTccagAG-3’ | 68.9 | -4.2 |
| 4SAMRS-3N | 5’-CCAATGCCAACATCTACCtccaGAG-3’ | 70.0 | -3.2 |
| 4SAMRS-4N | 5’-CCAATGCCAACATCTACctccAGAG-3’ | 69.0 | -4.1 |

| **Name** | **Sequence (5'- to 3')** | **T_m_ (^o^C)** | **ΔT_m_ (^o^C)** |
| --- | --- | --- | --- |
| 9-Std-Fp-25 | 5’-AAGGTTACGAAGTGCGCATCCTGAC-3’ | 75.2 | 0.0 |
| 2SAMRS-cTg-2N | 5’-AAGGTTACGAAGTGCGCATCcTgAC-3’ | 72.3 | -2.9 |
| 2SAMRS-1N | 5'-AAGGTTACGAAGTGCGCATCCTgaC-3` | 74.1 | -1.1 |
| 3SAMRS-1N | 5'-AAGGTTACGAAGTGCGCATCCtgaC-3` | 74.3 | -0.9 |
| 4SAMRS-1N | 5'-AAGGTTACGAAGTGCGCATCctgaC-3` | 73.7 | -1.5 |
| 4SAMRS-2N | 5'-AAGGTTACGAAGTGCGCATcctgAC-3` | 72.4 | -2.8 |
| 4SAMRS-3N | 5'-AAGGTTACGAAGTGCGCAtcctGAC-3` | 72.9 | -2.3 |
| 4SAMRS-4N | 5'-AAGGTTACGAAGTGCGCatccTGAC-3` | 72.5 | -2.7 |
|  |  |  |  |
| 13-Std-Rp-25 | 5'-GTTATCAGATTCGTCACCGGTCAGG-3` | 73.0 | 0.0 |
| 2SAMRS-cAg-1N | 5'-GTTATCAGATTCGTCACCGGTcAgG-3` | 70.3 | -2.7 |
| 2SAMRS-1N | 5'-GTTATCAGATTCGTCACCGGTCagG-3` | 71.5 | -1.5 |
| 3SAMRS-1N | 5'-GTTATCAGATTCGTCACCGGTcagG-3` | 70.6 | -2.5 |
| 4SAMRS-1N | 5'-GTTATCAGATTCGTCACCGGtcagG-3` | 71.0 | -2.0 |
| 4SAMRS-2N | 5'-GTTATCAGATTCGTCACCGgtcaGG-3` | 69.8 | -3.3 |
| 4SAMRS-3N | 5'-GTTATCAGATTCGTCACCggtcAGG-3` | 66.2 | -6.8 |
| 4SAMRS-4N | 5'-GTTATCAGATTCGTCACcggtCAGG-3` | 66.9 | -6.1 |

| **Name** | **Sequence (5`- to 3`)** | **T_m_ (^o^C)** | **ΔT_m_ (^o^C)** |
| --- | --- | --- | --- |
| 21-Std-Fp-23 | 5`-AGCTCTGCCCAAAGATTACCCTG-3` | 72.1 | 0.0 |
| 2SAMRS-cCc-2N | 5`-AGCTCTGCCCAAAGATTAcCcTG-3` | 69.0 | -3.1 |
| 2SAMRS-cc-3N | 5`-AGCTCTGCCCAAAGATTAccCTG-3` | 68.9 | -3.2 |
| 3SAMRS-acCc-2N | 5`-AGCTCTGCCCAAAGATTacCcTG-3` | 68.4 | -3.7 |
| 2SAMRS-1N | 5`-AGCTCTGCCCAAAGATTACCctG-3` | 71.1 | -1.0 |
| 3SAMRS-1N | 5`-AGCTCTGCCCAAAGATTACcctG-3` | 69.4 | -2.7 |
| 4SAMRS-1N | 5`-AGCTCTGCCCAAAGATTAccctG-3` | 67.7 | -4.4 |
| 4SAMRS-2N | 5`-AGCTCTGCCCAAAGATTacccTG-3` | 66.5 | -5.6 |
| 4SAMRS-3N | 5`-AGCTCTGCCCAAAGATtaccCTG-3` | 68.9 | -3.2 |
| 4SAMRS-4N | 5`-AGCTCTGCCCAAAGAttacCCTG-3` | 71.6 | -0.5 |
|  |  |  |  |
| 25-Std-Fp-25 | 5`-CCTCAGAAGTAGTGGCCAGCTGTGT-3` | 75.4 | 0.0 |
| 2SAMRS-gTg-1N | 5`-CCTCAGAAGTAGTGGCCAGCTgTgT-3` | 72.3 | -3.1 |
| 3SAMRS-cTgTg-1N | 5`-CCTCAGAAGTAGTGGCCAGcTgTgT-3` | 71.4 | -4.0 |
| 2SAMRS-cTg-3N | 5`-CCTCAGAAGTAGTGGCCAGcTgTGT-3` | 71.5 | -3.9 |
| 3SAMRS-gcTg-3N | 5`-CCTCAGAAGTAGTGGCCAgcTgTGT-3` | 69.2 | -6.2 |
| 4SAMRS-1N | 5`-CCTCAGAAGTAGTGGCCAGCtgtgT-3` | 73.1 | -2.3 |
| 4SAMRS-2N | 5`-CCTCAGAAGTAGTGGCCAGctgtGT-3` | 72.5 | -3.0 |
| 4SAMRS-3N | 5`-CCTCAGAAGTAGTGGCCAgctgTGT-3` | 69.9 | -5.6 |
| 4SAMRS-4N | 5`-CCTCAGAAGTAGTGGCCagctGTGT-3` | 71.2 | -4.2 |

| **Name** | **Sequence (5`- to 3`)** | **T_m_ (^o^C)** | **ΔT_m_ (^o^C)** |
| --- | --- | --- | --- |
| 17-Std-Rp-23 | 5`-CTGAGCTTCATGCCTTTACTGTT-3` | 69.7 | 0.0 |
| 1SAMRS-g-2N | 5`-CTGAGCTTCATGCCTTTACTgTT-3` | 67.4 | -2.3 |
| 2SAMRS-cTg-2N | 5`-CTGAGCTTCATGCCTTTAcTgTT-3` | 65.1 | -4.6 |
| 2SAMRS-1N | 5`-CTGAGCTTCATGCCTTTACTgtT-3` | 68.7 | -1.0 |
| 3SAMRS-1N | 5`-CTGAGCTTCATGCCTTTACtgtT-3` | 69.6 | -0.1 |
| 4SAMRS-1N | 5`-CTGAGCTTCATGCCTTTActgtT-3` | 66.7 | -3.0 |
| 4SAMRS-2N | 5`-CTGAGCTTCATGCCTTTactgTT-3` | 66.9 | -2.9 |
| 4SAMRS-3N | 5`-CTGAGCTTCATGCCTTtactGTT-3` | 68.9 | -0.8 |
| 4SAMRS-4N | 5`-CTGAGCTTCATGCCTttacTGTT-3` | 69.1 | -0.6 |
|  |  |  |  |
| 29-Std-Rp-23 | 5`-CTGAGCTTCATGCCTTTACTGTC-3` | 70.1 | 0.0 |
| 1SAMRS-g-2N | 5`-CTGAGCTTCATGCCTTTACTgTC-3` | 67.4 | -2.7 |
| 2SAMRS-cTg-2N | 5`-CTGAGCTTCATGCCTTTAcTgTC-3` | 65.3 | -4.8 |
| 4SAMRS-1N | 5`-CTGAGCTTCATGCCTTTActgtC-3` | 68.2 | -1.9 |
| 4SAMRS-2N | 5`-CTGAGCTTCATGCCTTTactgTC-3` | 66.7 | -3.4 |
| 4SAMRS-3N | 5`-CTGAGCTTCATGCCTTtactGTC-3` | 69.2 | -0.9 |
| 4SAMRS-4N | 5`-CTGAGCTTCATGCCTttacTGTC-3` | 69.3 | -0.9 |

**Supplementary Figure S2**

**Prevention of primer dimer in the worst-case scenario primer pairs**

Standard primers have six perfectly complementary overlaps of base pairs at 3'-end (underline sequences):

Std-Fp-25: 5’-AAGGTTACGAAGTGCGCATCCTGAC-3’

Std-Rp-25: 3'-GGACTGGCCACTGCTTAGACTATTG-5’

Fp-2SAMRS-1N 5'-AAGGTTACGAAGTGCGCATCCTgaC-3'

Rp-2SAMRS-1N 3'-GgaCTGGCCACTGCTTAGACTATTG-5’

Fp-3SAMRS-1N 5'-AAGGTTACGAAGTGCGCATCCtgaC-3'

Rp-3SAMRS-1N 3'-GgacTGGCCACTGCTTAGACTATTG-5’

Fp-4SAMRS-1N 5'-AAGGTTACGAAGTGCGCATCctgaC-3'

Rp-4SAMRS-1N 3'-GgactGGCCACTGCTTAGACTATTG-5’

Fp-4SAMRS-2N 5'-AAGGTTACGAAGTGCGCATcctgAC-3'

Rp-4SAMRS-2N 3'-GGactgGCCACTGCTTAGACTATTG-5`

Fp-4SAMRS-3N 5'-AAGGTTACGAAGTGCGCAtcctGAC-3'

Rp-4SAMRS-3N 3'-GGActggCCACTGCTTAGACTATTG-5`

Fp-4SAMRS-4N 5'-AAGGTTACGAAGTGCGCatccTGAC-3'

Rp-4SAMRS-4N 3'-GGACtggcCACTGCTTAGACTATTG-5`

6pair-psW47Taq-110

5`-AAGGTTACGAAGTGCGCATCCTGACTGCGGACAAAGATCTGTACCAGCTGCTGTCTGACCGTGA

TCAGTGGGCTGACTACCGCGCCCTGACCGGTGACGAATCTGATAAC-3`

Std-Fp-25 5’-AAGGTTACGAAGTGCGCATCCTGAC -3’

Rp-2SAMRS-1N 3’-GgaCTGGCCACTGCTTAGACTATTG-5’

Rp-3SAMRS-1N 3’-GgacTGGCCACTGCTTAGACTATTG-5’

Rp-4SAMRS-1N 3’-GgactGGCCACTGCTTAGACTATTG-5’

Rp-4SAMRS-2N 3’-GGactgGCCACTGCTTAGACTATTG-5'

Rp-4SAMRS-3N 3’-GGActggCCACTGCTTAGACTATTG-5'

Rp-4SAMRS-4N 3’-GGACtggcCACTGCTTAGACTATTG-5'

**Supplementary Figure S2.** Melting curves of amplicon and primer dimers generated by standard forward primer and different SAMRS reverse primers having six perfectly matched base pairs at their 3'-ends (a "worst case" pair of primers).

**Left**: different numbers (2, 3, and 4) of SAMRS components are placed one nucleotide (1N) close to the 3'-end of the reverse primer.

**Right**: four SAMRS components are placed 1, 2, 3, and 4 nucleotides away from the 3'-end of the reverse primer (1N, 2N, 3N, and 4N).

Melting curves with target (Tgt, solid lines) or without target (NTC, dashed lines).

The forward primer is entirely standard, the reverse primer contains SAMRS components in different numbers (**left panel**) and with different placement (**right panel**). The desired amplicon has T_m_ of ~88.5 °C; for the undesired primer dimer, T_m_ < 82°C (depending on the numbers and positions of SAMRS).

From the left panel, note how suppression of primer dimer is greatest with reverse primers containing more SAMRS, red > blue > green > black, respectively 4, 3, 2, and 0 SAMRS components, the last (black) showing essentially only primer dimer.

From the right panel, note how suppression of primer dimer is greatest in the pairs with 4SAMRS one nucleotide (1N) closest to the 3'-end of the reverse primer, red > aqua > violet > green, respectively 1, 2, 3, and 4 nucleotides close to the 3`-end, the last showing essentially only primer dimer.

**Evaluating SAMRS-containing primers to support efficient PCR, suppress primer dimer, and improve single nucleotide polymorphisms (SNPs) discrimination**

Primers containing different numbers (1, 2, 3, or 4) of SAMRS components:

5’-TCCAAAGTAGCATGACAAAAATCTTAGAGC-3' HIV-Std-Fp

5’-TCCAAAGTAGCATGACAAAAATCTTAGA*A*C-3' Fp-1Mis-Gto*A*

5'-TCCAAAGTAGCATGACAAAAATCTTAGAgC-3' Fp-1SAMRS-g-1N

5'-TCCAAAGTAGCATGACAAAAATCTTAGagC-3' Fp-2SAMRS-ag-1N

5'-TCCAAAGTAGCATGACAAAAATCTTAgagC-3' Fp-3SAMRS-gag-1N

5`-TCCAAAGTAGCATGACAAAAATCTTagagC-3' Fp-4SAMRS-agag-1N

HIV-allele-A-Template

# 5'-TCCAAAGTAGCATGACAAAAATCTTAGAGCCTTTTAGAAAACAAAATCCAGACATAGTTATCTAT

# CAATACATGGATGATTTGTATGTAAGATCTGACTTAGAAATAGGGCAGCAT-3'

HIV-allele-G-Template

# 5'-TCCAAAGTAGCATGACAAAAATCTTAGAGCCTTTTAGAAAACAAAATCCAGACATAGTTATCTAT

# CAATACATGGATGATTTGTATGTAGGATCTGACTTAGAAATAGGGCAGCAT-3'

HIV-Std-Rp 3`-CCTAGACTGAATCTTTATCCCGTCGTA-5'

Rp-1Mis-Cto*T* 3`-C*T*TAGACTGAATCTTTATCCCGTCGTA-5'

Rp-1SAMRS-Tc-1N 3`-CcTAGACTGAATCTTTATCCCGTCGTA-5'

Rp-2SAMRS-tc-1N 3`-CctAGACTGAATCTTTATCCCGTCGTA-5'

Rp-3SAMRS-atc-1N 3`-CctaGACTGAATCTTTATCCCGTCGTA-5'

Rp-4SAMRS-gatc-1N 3`-CctagACTGAATCTTTATCCCGTCGTA-5'

Rp-2SAMRS-aTc-1N 3`-CcTaGACTGAATCTTTATCCCGTCGTA-5'

Rp-3SAMRS-gaTc-1N 3`-CcTagACTGAATCTTTATCCCGTCGTA-5'

**Supplementary Table S4-1.**

Ct and ΔCt values of PCR amplifications using forward primer with different numbers of SAMRS components with KlenTaq1 DNA polymerase.

| Allele-specific PCR using KlenTaq1 DNA Polymerase | | | | |
| --- | --- | --- | --- | --- |
| Forward primer | Reverse primer | Match C:G (ΔCt) | Mismatch C:A (ΔCt) | NTC (ΔCt) |
| HIV-Std-Fp | Common HIV-Std-Rp | 29.6 (0) reference | 33.7 (4.1) | 39.8 (10.2) |
| Std-1Mis (G to *A*) |  | 29.8 (0.2) | 35.5 (5.9, dimer) | 33.1 (3.5) |
| 1SAMRS-g-1N |  | 30.6 (1) | 35.6 (6) | 37.5 (7.9)** |
| 2SAMRS-ag-1N |  | 30.6 (1) | 37.8 (8.2, dimer) | 39.1 (9.5) |
| 3SAMRS-gag-1N |  | 31 (1.4) | 41.8 (12.2, dimer) | 43.2 (13.6)* |
| 4SAMRS-agag-1N |  | 35.7 (6.1) | 45.9 (16.3)* | 46.5 (16.9)* |

A "least-worst case" pair of primers was tested in allele-specific PCR (60 cycles).

All PCR have three replicates for matched template G, mismatched template A, and no template control (NTC). * indicates 1/3, ** indicate 2/3 of replicates show amplification signals.

Dimer indicates primer dimer. NA indicates no amplification.

**Supplementary Table S4-2.**

Ct and ΔCt values of PCR amplifications using forward and reverse primers with different numbers of SAMRS components with KlenTaq1 DNA polymerase.

| Allele-specific PCR using KlenTaq1 DNA Polymerase | | | | |
| --- | --- | --- | --- | --- |
| Forward Primer | Reverse Primer | Match C:G (ΔCt) | Mismatch C:A (ΔCt) | NTC (ΔCt) |
| HIV-Std-Fp | HIV-Std-Rp | 29.9 (0) reference | 32.9 (3.0) | 33.7 (3.8) |
| Std-1Mis (G to *A*) | Std-1Mis (C to *T*) | 30.6 (0.7) | 34.3 (4.4, dimer) | 33.9 (4.0) |
| 1SAMRS-g-1N | 1SAMRS-Tc-1N | 31 (1.1) | 34.4 (4.5) | 36.7 (6.8)* |
| 2SAMRS-ag-1N | 2SAMRS-tc-1N | 31 (1.1) | 35.3 (5.4) | NA |
| 3SAMRS-gag-1N | 3SAMRS-atc-1N | 33 (3.1) | 42.7 (12.8) | NA |
| 4SAMRS-agag-1N | 4SAMRS-gatc-1N | 40.3 (10.4) | 51.9 (22.0) | NA |

A "least-worst case" pair of primers was tested in allele-specific PCR (60 cycles).

All PCR have three replicates for matched template G, mismatched template A, and no template control (NTC). * indicates 1/3, ** indicate 2/3 of replicates show amplification signals.

Dimer indicates primer dimer. NA indicates no amplification.

**Evaluating different numbers and positions of SAMRS components in primer**

Common Std-Fp-23

5`-AGCTCTGCCCAAAGATTACCCTG-3`

Std-Rp-T-allele 3`-TTGTCATTTCCGTACTTCGAGTC-5`

5`-AGCTCTGCCCAAAGATTACCCTGACAGCTAAGTGGCAGTGGAAGTTGGCCTCAGAAGTAGTGGCCAGCTGTGTGTCGGGG**A**ACAGTAAAGGCATGAAGCTCAG-3`

Allele-A-Template

5`-AGCTCTGCCCAAAGATTACCCTGACAGCTAAGTGGCAGTGGAAGTTGGCCTCAGAAGTAGTGGCCAGCTGTGTGTCGGGG**G**ACAGTAAAGGCATGAAGCTCAG-3`

Allele-G-Template

5`-AGCTCTGCCCAAAGATTACCCTGACAGCTAAGTGGCAGTGGAAGTTGGCCTCAGAAGTAGTGGCCAGCTGTGTGTCGGGG**C**ACAGTAAAGGCATGAAGCTCAG-3`

Allele-C-Template

5`-AGCTCTGCCCAAAGATTACCCTGACAGCTAAGTGGCAGTGGAAGTTGGCCTCAGAAGTAGTGGCCAGCTGTGTGTCGGGG**T**ACAGTAAAGGCATGAAGCTCAG-3`

Allele-T-Template

**Std-Rp-T-allele:** 5`-CTGAGCTTCATGCCTTTACTGTT-3` 3`-TTGTCATTTCCGTACTTCGAGTC-5`

Rp-2SAMRS-Tgt-1N 5`-CTGAGCTTCATGCCTTTACTgtT-3` 3`-TtgTCATTTCCGTACTTCGAGTC-5`

Rp-3SAMRS-tgt-1N 5`-CTGAGCTTCATGCCTTTACtgtT-3` 3`-TtgtCATTTCCGTACTTCGAGTC-5`

Rp-4SAMRS-ctgt-1N 5`-CTGAGCTTCATGCCTTTActgtT-3` 3`-TtgtcATTTCCGTACTTCGAGTC-5`

Rp-4SAMRS-actg-2N 5`-CTGAGCTTCATGCCTTTactgTT-3` 3`-TTgtcaTTTCCGTACTTCGAGTC-5`

Rp-4SAMRS-tact-3N 5`-CTGAGCTTCATGCCTTtactGTT-3` 3`-TTGtcatTTCCGTACTTCGAGTC-5`

Rp-4SAMRS-ttac-4N 5`-CTGAGCTTCATGCCTttacTGTT-3` 3`-TTGTcattTCCGTACTTCGAGTC-5`

Rp-2SAMRS-cTgT-2N 5`-CTGAGCTTCATGCCTTTAcTgTT-3` 3`-TTgTcATTTCCGTACTTCGAGTC-5`

**Std-Rp-C-allele:** 5`-CTGAGCTTCATGCCTTTACTGTC-3` 3`-CTGTCATTTCCGTACTTCGAGTC-5`

Rp-4SAMRS-ctgt-1N 5`-CTGAGCTTCATGCCTTTActgtC-3` 3`-CtgtcATTTCCGTACTTCGAGTC-5`

Rp-4SAMRS-actg-2N 5`-CTGAGCTTCATGCCTTTactgTC-3` 3`-CTgtcaTTTCCGTACTTCGAGTC-5`

Rp-4SAMRS-tact-3N 5`-CTGAGCTTCATGCCTTtactGTC-3` 3`-CTGtcatTTCCGTACTTCGAGTC-5`

Rp-4SAMRS-ttac-4N 5`-CTGAGCTTCATGCCTttacTGTC-3` 3`-CTGTCATTTCCGTACTTCGAGTC-5`

Rp-2SAMRS-cTgT-2N 5`-CTGAGCTTCATGCCTTTAcTgTC-3` 3`-CTgTcATTTCCGTACTTCGAGTC-5`

**Supplementary Table S5-1.**

Ct and ΔCt values of PCR amplifications using reverse primer with different positions of SAMRS components with KlenTaq1 DNA polymerase.

| Ct and ΔCt of SNP discrimination using KlenTaq1 DNA Polymerase | | | | | | | |
| --- | --- | --- | --- | --- | --- | --- | --- |
| Forward Primer | Reverse Primer | Match C:G | Mismatch C:A | Mismatch C:C | Mismatch C:T | Average ΔCt | NTC |
| Common Std-Fp | Std-Rp-C-allele | 29.9 (0) | 33.3 (3.4) | 43.2 (13.3) | 31.0 (1.1) | (6.0) | NA |
|  | 4SAMRS-1N | 32.4 (2.5) | 41.4 (11.5) | 40 (10.1)* (dimer) | 39.1 (9.2) | (10.4) | NA |
|  | 4SAMRS-2N | 33.1 (3.2) | 42.8 (12.9) | 41 (11.1)* (dimer) | 40.7 (10.8) | (11.7) | NA |
|  | 4SAMRS-3N | 31.9 (2) | 39.1 (9.2) | 39.6 (9.7)* (dimer) | 36.8 (6.9) | (8.6) | NA |
|  | 4SAMRS-4N | 32.9 (3) | 39.6 (9.7) | 46.8 (16.9) | 37.4 (7.5) | (11.5) | NA |

**Supplementary Table S5-2.**

Ct and ΔCt values of PCR amplifications using reverse primer with different positions of SAMRS components with HiDi DNA polymerase.

| Ct and ΔCt of SNP discrimination using HiDi DNA Polymerase | | | | | | |
| --- | --- | --- | --- | --- | --- | --- |
| Forward Primer | Reverse Primer | Match C:G | Mismatch C:A | Mismatch C:C | Mismatch C:T | NTC |
| Common Std-Fp | Std-Rp-C-allele | 28.6 (0) | 33.8 (5.2) | NA | 41.9 (13.3)* | NA |
|  | 4SAMRS-1N | 34.0 (5.4) | 42.0 (13.4) | NA | NA | NA |
|  | 4SAMRS-2N | 35.6 (7) | 43.9 (15.3) | NA | NA | NA |
|  | 4SAMRS-3N | 32.7 (4.1) | 39.0 (10.4) | NA | NA | NA |
|  | 4SAMRS-4N | 34.0 (5.4) | 41.1 (12.5) | NA | NA | NA |

All allele-specific PCR have three replicates for matched template G, mismatched templates (A, C, and T), and no template control (NTC). * indicates 1/3, ** indicate 2/3 of replicates show amplification signals.

Dimer indicates primer dimer. NA indicates no amplification.

**Evaluation of SAMRS components: t and consecutive c**'s

Std-Fp-23

5`-AGCTCTGCCCAAAGATTACCCTG-3`

Std-Rp-T-allele 3`-TTGTCATTTCCGTACTTCGAGTC-5'

5`-AGCTCTGCCCAAAGATTACCCTGACAGCTAAGTGGCAGTGGAAGTTGGCCTCAGAAGTAGTGGCCAGCTGTGTGTCGGGG**A**ACAGTAAAGGCATGAAGCTCAG-3'

Allele-A-Template

Std-Fp-23 5`-AGCTCTGCCCAAAGATTACCCTG-3'

4SAMRS-ccct-1N 5`-AGCTCTGCCCAAAGATTAccctG-3'

4SAMRS-accc-2N 5`-AGCTCTGCCCAAAGATTacccTG-3'

2SAMRS-cCcT-2N 5`-AGCTCTGCCCAAAGATTAcCcTG-3'

3SAMRS-acCc-2N 5`-AGCTCTGCCCAAAGATTacCcTG-3'

Std-Fp-25 5`-CCTCAGAAGTAGTGGCCAGCTGTGT-3'

4SAMRS-tgtg-1N 5`-CCTCAGAAGTAGTGGCCAGCtgtgT-3'

4SAMRS-ctgt-2N: 5`-CCTCAGAAGTAGTGGCCAGctgtGT-3'

4SAMRS-gctg-3N: 5`-CCTCAGAAGTAGTGGCCAgctgTGT-3'

2SAMRS-TgTg-1N 5`-CCTCAGAAGTAGTGGCCAGCTgTgT-3'

2SAMRS-cTgT-3N 5`-CCTCAGAAGTAGTGGCCAGcTgTGT-3'

3SAMRS-gcTg-3N 5`-CCTCAGAAGTAGTGGCCAgcTgTGT-3'

**Supplementary Table S6-1.**

Compare primers with or without SAMRS component "**t**"

| Ct and ΔCt of different SAMRS primers with or without "t" | | | |
| --- | --- | --- | --- |
| Forward Primer | Reverse Primer | Match T:A | NTC |
| Std-Fp-25 | Common Std-Rp-T-allele | 27.6 (0.0) | 41.6 (14) |
| 4SAMRS-tgtg-1N |  | 27.8 (0.2) | 43.3 (15.7) |
| 4SAMRS-ctgt-2N |  | 27.9 (0.3) | 42.6 (15) |
| 4SAMRS-gctg-3N |  | 28.1 (0.5) | 42.7 (15.1) |
| 2SAMRS-TgTg-1N |  | 27.6 (0.0) | 47.5 (19.9)** |
| 2SAMRS-cTgT-3N |  | 27.6 (0.0) | 46.4 (18.8)** |
| 3SAMRS-gcTg-3N |  | 27.9 (0.3) | 51.5 (23.9)* |

* indicates 1/3 and ** indicate 2/3 of replicates show amplification signal.

**Supplementary Table S6-2.**

Compare SAMRS primers with or without consecutive **c**'s

| Ct and ΔCt of different SAMRS primers with or without consecutive c's | | | |
| --- | --- | --- | --- |
| Forward Primer | Reverse Primer | Match T:A | NTC |
| Std-Fp-23 | Common Std-Rp-T-allele | 28.8 (0) | 38.5 (9.7) |
| 4SAMRS-ccct-1N |  | 29.6 (0.8) | NA |
| 4SAMRS-accc-2N |  | 30.3 (1.5) | 55* |
| 2SAMRS-cCcT-2N |  | 29.4 (0.6) | NA |
| 3SAMRS-acCc-2N |  | 29.6 (0.8) | NA |

*1/3 replicates gave signal, NA indicates no amplification.
